# Genomic evidence for domestication selection in three hatchery populations of Chinook salmon, *Oncorhynchus tshawytscha*

**DOI:** 10.1111/eva.13656

**Published:** 2024-02-14

**Authors:** Natasha S. Howe, Matthew C. Hale, Charles D. Waters, Sara M. Schaal, Kyle R. Shedd, Wesley A. Larson

**Affiliations:** ^1^ Department of Biology Texas Christian University Fort Worth Texas USA; ^2^ National Oceanographic and Atmospheric Administration, National Marine Fisheries Service Alaska Fisheries Science Center, Auke Bay Laboratories Juneau Alaska USA; ^3^ Alaska Department of Fish and Game, Division of Commercial Fisheries Gene Conservation Laboratory Anchorage Alaska USA

**Keywords:** domestication selection, hatchery, low‐coverage whole‐genome sequencing, rapid adaptation, salmon, Southeast Alaska

## Abstract

Fish hatcheries are widely used to enhance fisheries and supplement declining wild populations. However, substantial evidence suggests that hatchery fish are subject to differential selection pressures compared to their wild counterparts. Domestication selection, or adaptation to the hatchery environment, poses a risk to wild populations if traits specific to success in the hatchery environment have a genetic component and there is subsequent introgression between hatchery and wild fish. Few studies have investigated domestication selection in hatcheries on a genomic level, and even fewer have done so in parallel across multiple hatchery–wild population pairs. In this study, we used low‐coverage whole‐genome sequencing to investigate signals of domestication selection in three separate hatchery populations of Chinook salmon, *Oncorhynchus tshawytscha*, after approximately seven generations of divergence from their corresponding wild progenitor populations. We sequenced 192 individuals from populations across Southeast Alaska and estimated genotype likelihoods at over six million loci. We discovered a total of 14 outlier peaks displaying high genetic differentiation (*F*_ST_) between hatchery–wild pairs, although no peaks were shared across the three comparisons. Peaks were small (53 kb on average) and often displayed elevated absolute genetic divergence (*D*_*xy*_) and linkage disequilibrium, suggesting some level of domestication selection has occurred. Our study provides evidence that domestication selection can lead to genetic differences between hatchery and wild populations in only a few generations. Additionally, our data suggest that population‐specific adaptation to hatchery environments likely occurs through different genetic pathways, even for populations with similar standing genetic variation. These results highlight the need to collect paired genotype–phenotype data to understand how domestication may be affecting fitness and to identify potential management practices that may mitigate genetic risks despite multiple pathways of domestication.

## 1 INTRODUCTION

Our understanding of adaptation is expanding as genomics increases our power to uncover the genetic basis of phenotypic variation and how it may respond to environmental change (Bomblies & Peichel, 2022). Adaptation is generally thought to occur over hundreds or thousands of generations, yet recent evidence shows that it can also happen on much shorter timescales (Rudman et al., 2022; Van't Hof et al., 2011). This has been observed when species either undergo adaptive radiation to fill various ecological niches (Grant & Grant, 2002) or when a novel, strong selection pressure is introduced to a population (Ravinet et al., 2016; Therkildsen et al., 2019). In some cases, multiple independent populations have been subjected to rapidly changing environments with analogous selection pressures, and they responded by adapting at the same genomic regions (Colosimo et al., 2005; Winchell et al., 2023; Zong et al., 2020). However, we still do not have a thorough understanding of how species and populations respond to shared selective pressures with parallel genomic adaptations. As anthropogenic activities create novel environments to which species must rapidly adapt (Palumbi, 2001), such insights may help develop targeted conservation strategies that can facilitate the preservation of genetic diversity in vulnerable species.

Rapid adaptation is particularly common during domestication, when selection pressures differ substantially from those in the wild (Venney et al., 2021). Domestication is a human‐mediated intervention that can cause relaxation of natural selection and introduce artificial selection pressures to individuals in the captive environment (Balon, 2004; Mignon‐Grasteau et al., 2005). In many cases, domestication selection for certain traits is deliberate, such as increased milk production in dairy cattle (Flori et al., 2009), coat color in domestic pigs (Fang et al., 2009), and resistance to diseases in aquaculture facilities (Hillestad et al., 2020). Alternatively, the objective of captive breeding can be to introduce as little artificial selection as possible, as in the case of conservation‐focused breeding programs, which have gained increasing importance as more species become threatened and endangered (Schulte‐Hostedde & Mastromonaco, 2015). Although breeding programs are often designed to preserve genetic diversity, they can inadvertently cause divergence from their wild progenitors (Doublet et al., 2019; Luo et al., 2022). This can decrease the fitness of captive‐bred individuals when released in the wild (Blouin et al., 2021; O'Sullivan et al., 2020; Schubert et al., 2014). Therefore, identifying and reducing any detrimental effects of domestication selection is a crucial aspect of conservation.

Pacific salmon hatcheries are a type of captive breeding used in conservation management to supplement declining wild populations and enhance stocks for harvest (Amoroso et al., 2017). However, evidence suggests that hatchery‐rearing can inadvertently select for traits that may be disadvantageous in the wild, which can have subsequent implications for native stocks (Koch & Narum, 2021). Unlike most methods of captive breeding, hatchery‐reared salmon are released into the wild once they complete their freshwater juvenile life stage. During this juvenile stage in captivity, natural selection is relaxed, as fish are protected from predation and reared in a stable environment with abundant food. Hatchery‐rearing also deprives fish of environmental stimuli from complex habitats, such as large woody debris, and exposes them to novel stimuli (Mes et al., 2018). All of these modifications may affect life expectancy and behavior. For example, egg‐to‐smolt survival in hatcheries is commonly above 85% (Reisenbichler et al., 2004), compared to 1%–10% in the wild (Quinn, 2005). Additionally, hatchery fish show increased competitive behavior and dominance (Metcalfe et al., 2003; Wessel et al., 2006), changes in run timing (Ford et al., 2006), faster growth (Blouin et al., 2021; Fleming & Einum, 1997), and reduced predator avoidance compared to wild fish (Álvarez & Nicieza, 2003). Furthermore, when hatchery fish are released into the wild, they generally have reduced reproductive success (Christie et al., 2014; Koch & Narum, 2021; Thériault et al., 2011) and decreased survival rates (Beamish et al., 2012; Blouin et al., 2021; Christie et al., 2012) compared to their wild counterparts. Such divergence poses a risk to wild populations if hatchery‐reared individuals interbreed with wild individuals (Besnier et al., 2022; Grant, 2011; Hagen et al., 2019; Utter, 1998). Therefore, increased knowledge of the genetic pathways involved in hatchery domestication and the repeatability of those pathways would provide a greater understanding of how hatchery populations differ from their wild progenitors. This information can then be used to aid in the development of management approaches that reduce unwanted divergence.

While phenotypic differences between hatchery and wild salmon are consistently reported and widely accepted (Naish et al., 2007), the causative genetic basis and the corresponding genetic repeatability across independent hatchery–wild population pairs remain unresolved. Most genomic regions found to differ across hatchery and wild salmon have been specific to the study system (Ford et al., 2023; Waters et al., 2015, 2018). In Chinook salmon, Waters et al. (2015, 2018) observed that the genetic divergence of a segregated hatchery population from the founding wild population increased with each additional generation of hatchery rearing due to the combined effects of drift and domestication selection. Furthermore, the authors identified specific loci that diverged across consecutive generations of hatchery‐rearing and linked some of these loci to fitness‐related traits such as spawn timing, suggesting that genetic and phenotypic effects of domestication can occur at rapid timescales (Waters et al., 2015, 2018). In addition, a whole‐genome sequencing study on Chinook salmon identified divergence between hatchery and wild fish after at least one generation in captivity (Ford et al., 2023). These studies provide insightful genetic comparisons of wild and hatchery Pacific salmon, but it remains unclear if the genetic pathways of domestication selection are consistent across hatcheries.

In this study, we explored domestication selection by comparing three hatchery–wild population pairs of Chinook salmon in Southeast Alaska (SEAK) that have been separated for approximately seven generations (30–40 years). We used both low‐coverage whole‐genome sequencing (lcWGS) data and high‐coverage, low marker density data from a genotyping‐in‐thousands by sequencing (GT‐seq) panel to assess population structure, diversity, and effective population sizes. These pairwise comparisons were used to (i) discover genomic regions displaying high differentiation within each hatchery population compared to their wild progenitor population and (ii) identify if there were shared regions of adaptive divergence across the three hatchery–wild pairwise comparisons. These results provide fine‐scale genomic evidence for domestication and highlight the need to assess if specific management practices, such as the integration of wild broodstock, can help mitigate genetic risks despite multiple pathways of domestication.

## 2 METHODS

### 2.1 Population descriptions

All three of the hatcheries in our study are segregated hatcheries intended to enhance commercial and recreational fisheries instead of supplementing wild populations. Each hatchery is located on the coast and separated from the founding wild population and other wild populations by approximately 70 to 220 km. Broodstock are collected from fish returning to each hatchery from the ocean, and introgression from wild or other hatchery stocks is assumed to be rare (see below). However, two of the hatcheries do not screen their broodstock (i.e. confirm the stock of origin), so introgression from stray wild fish or fish from other hatcheries may occur at a low rate. Juveniles are released as smolts directly into the ocean, either at the hatchery or at remote release locations away from wild populations (Wilson, 2023).

**FIGURE 1:**
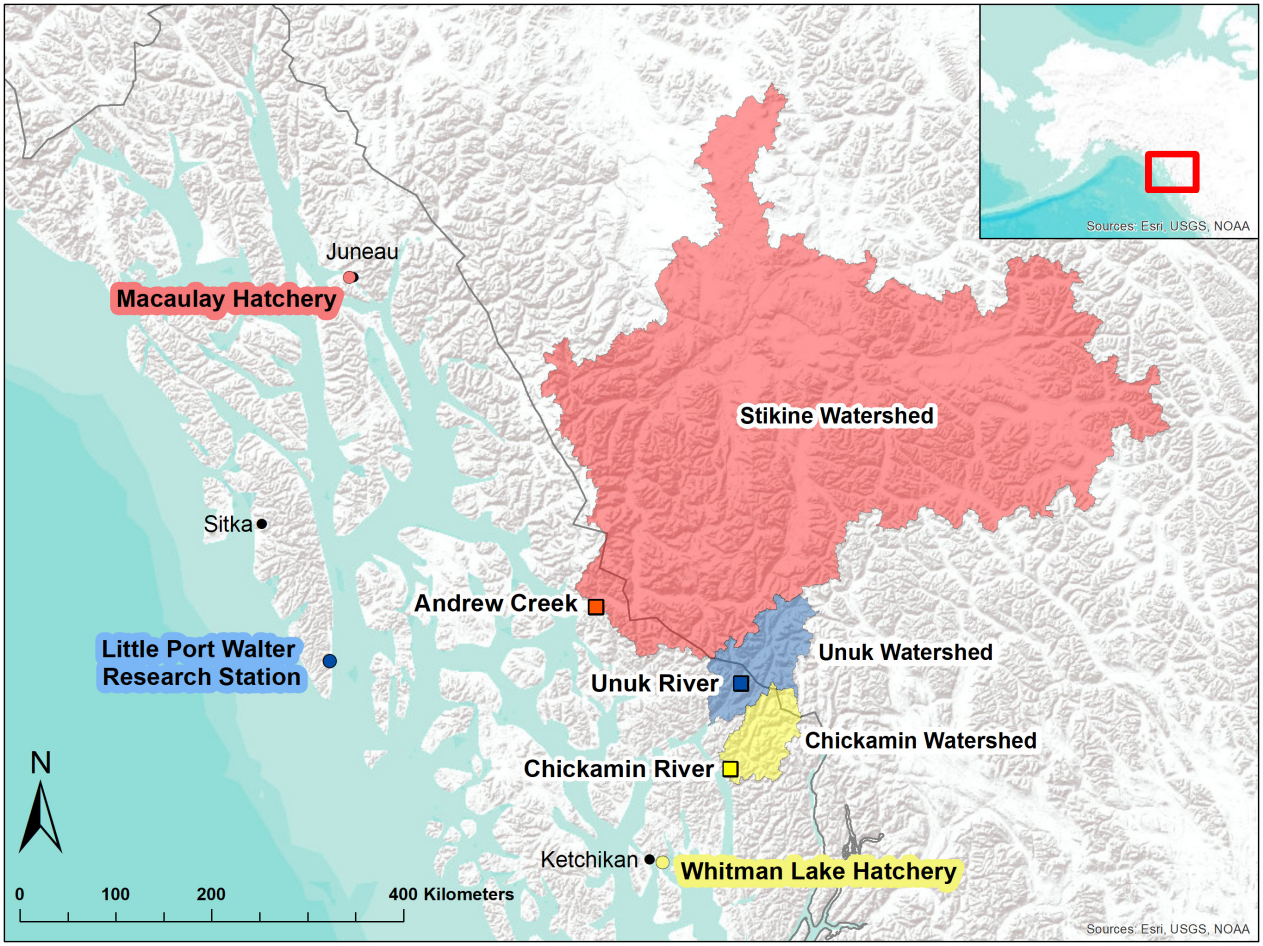
Site map of Southeast Alaska with the locations for each hatchery population (circle) and corresponding wild population (square), as well as the respective watersheds for each wild population. Matching colors are indicative of hatchery–wild population pairs, for which the wild population is the hatchery's progenitor stock.

The three hatchery populations in our study differ substantially in their fish culture methods and goals. Little Port Walter (LPW) is a research facility that maintains a hatchery line of Chinook salmon with smaller returns (one to two thousand) and broodstock sizes (100–200 fish in most years). In contrast, Whitman Lake Hatchery and Macaulay Hatchery are production‐focused hatcheries that produce larger numbers of fish (returns >10,000; broodstock sizes >400) to supplement commercial and recreational fisheries (L. Wilson (ADF&G), personal communication). Although all are functionally segregated hatcheries (no gene flow between wild and hatchery fish), some broodstock management techniques are unique to each facility that result in differing effective population sizes and potentially different selective pressures. More information on hatchery and wild populations is found below, as well as in Figure 1 and Table 1.

**TABLE 1:** Hatchery and wild population pairs, sampling year and sample sizes for lcWGS and GT‐seq samples, average annual escapement to wild streams and returns to hatcheries, estimated effective population sizes (*N*_e_) with corresponding parametric confidence intervals, observed heterozygosity from lcWGS and GT‐seq (*H*_o_), and expected heterozygosity (*H*_E_) from GT‐seq.

Pair	Site	Sample year	WGS *n*	GT‐seq *n*	Returns^b^/escapement	*N*_e_ (CI)	WGS H_o_ (SE)	GT‐seq *H*_o_ (SE)	GT‐seq *H*_E_ (SE)
1	Unuk River (Wild)	1988	16	95	1849	1397 (679–∞)	0.298 (0.00006)	0.277 (0.012)	0.277 (0.011)
		2004	16	95					
	Little Port Walter (Hatchery)	2020	32	46^a^	736	92 (78–109)	0.293 (0.00006)	0.280 (0.012)	0.277 (0.011)
2	Andrew Creek (Wild)	1989	16	0	690	1582 (1010–3485)	0.296 (0.00005)	0.272 (0.011)	0.273 (0.011)
		2004	16	188					
	Macaulay (Hatchery)	2014	32	46	2150	535 (288–2895)	0.299 (0.00005)	0.269 (0.012)	0.266 (0.012)
3	Chickamin River (Wild)	1990	16	0	2187	349 (274–477)	0.292 (0.00007)	0.279 (0.012)	0.277 (0.011)
		2005	16	91					
	Whitman Lake (Hatchery)	2014	32	47	5323	264 (188–441)	0.302 (0.00006)	0.270 (0.012)	0.272 (0.012)

^a^ Collection year was 2018.

^b^ Returns = number of Chinook returns to hatchery; Escapement = number of Chinook returns to river to spawn. Values represent 10‐year average up to the most recent reporting. Escapement data from Meredith et al. (2022) and Salomone et al. (2022). Hatchery return data from L. Wilson (ADF&G), personal communication.

LPW Research Station is operated by the National Oceanic and Atmospheric Administration and is located on southeastern Baranof Island, Alaska (Figure 1). The LPW hatchery line included in this study was started in 1976 (Moberly & Kaill, 1977; Templin, 2001) and is derived from Cripple Creek on the Unuk River, located near Ketchikan, Alaska. Unuk River has an average annual escapement (i.e., number of fish returning to streams to spawn) of approximately 1800 adults in the past ten years (Meredith et al., 2022). Wild broodstock from the Unuk River were collected annually from 1976–1981 to initiate the LPW research hatchery stock (total of 128 females and 119 males; Templin, 2001). Unuk River wild gametes from nine males and nine females were also infused into the LPW research hatchery stock in 1998 (Templin, 2001). Each year, LPW releases an average of 107,000 tagged Unuk smolts (nearly 100% tagging rate) and collects all returning adults (average return = 1154, SD = 968) to propagate the next generation (L. Wilson (ADF&G), personal communication). Only tagged LPW fish from the Unuk stock were used to spawn the following generation (average broodstock = 171 over the last decade), and matings usually entailed evenly splitting the eggs from one female and fertilizing by two males.

Whitman Lake Hatchery is a production‐focused facility located in Ketchikan, Alaska (Figure 1) and operated by the Southern Southeast Regional Aquaculture Association (SSRAA). Whitman Lake broodstock is derived from the Chickamin River, also near Ketchikan, which had an average annual escapement of approximately 2000 adults in the last ten years (Meredith et al., 2022). Whitman Lake Hatchery began in 1981 by transferring eggs derived from a LPW hatchery stock of South Fork Chickamin salmon, which was initiated in 1976 (i.e., eggs from returning first‐generation Chickamin hatchery fish; Moberly & Kaill, 1977; Templin, 2001). Whitman Lake received additional hatchery‐origin Chickamin eggs and fry from other facilities in 1987, 1993, 1994, and 2013 (L. Wilson (ADF&G), personal communication). Wild broodstock was also collected annually from King Creek and Barrier Creek of the Chickamin River from 1983–1987 for broodstock (total of 204 females and 104 males; Amend, 1987; Templin, 2001). An average of 663,000 Chickamin stock smolts have been released annually from Whitman Lake over the past 10 years, with approximately 13% of the fish coded‐wire tagged (RMIS, 1977). Matings at Whitman Lake are typically one female fertilized by two males. The facility collects gametes (average brood size = 865 for the last 10 years) from returning adults (average return = 5671, SD = 2515 over the last decade) to produce the next generation (L. Wilson (ADF&G), personal communication; Tessa Frost (SSRAA), personal communication); however, the origin of most adults cannot be determined due to low tagging rates and no broodstock screening. Therefore, there is the possibility that stray wild and hatchery fish from other stocks are occasionally spawned.

Macaulay Hatchery is a production‐focused hatchery operated by Douglas Island Pink and Chum (DIPAC) in Juneau, Alaska (Figure 1). The wild progenitor population of the Macaulay hatchery line is Andrew Creek, a tributary of the lower Stikine River. Andrew Creek had an average annual escapement of 690 adults over the past 10 years (Salomone et al., 2022). The Andrew Creek hatchery stock was initiated at another facility from 1976 to 1982 when gametes were collected annually from wild broodstock (approximate total of 332 females and 233 males; L. Wilson (ADF&G), personal communication; Mecum, 1990; Templin, 2001). Hatchery‐origin eggs and juveniles were transferred from 1987–1992 to initiate production at Macaulay (formerly known as Gastineau Hatchery; Templin, 2001). The facility collects gametes (average of 430 broodstock over the last 10 years) from returning adults to produce the next generation (L. Wilson (ADF&G), personal communication), although additional inputs of Andrew Creek hatchery stock from other facilities have been received by Macaulay in some years (L. Wilson (ADF&G), personal communication; see Templin, 2001 for details). Matings at Macaulay are typically one female fertilized by two to four males (K. Harms (DIPAC), personal communication). An average of 834,000 Andrew Creek stock smolts have been released annually from Macaulay and nearby locations over the past ten years, with approximately 14% of the fish coded‐wire tagged (RMIS, 1977). Returns to Macaulay Hatchery averaged 2150 (SD = 1649) annually over the last decade (L. Wilson (ADF&G), personal communication). Like Whitman Lake Hatchery, the origin of most of the adults returning to Macaulay cannot be determined since, until recently, only a fraction of the released fish were tagged. Therefore, there is the possibility that stray wild and hatchery fish from other stocks are occasionally spawned.

### 2.2 Sample collection

Fin clips were collected from returning adult Chinook salmon at hatchery facilities, and samples from wild populations were collected during spawning ground surveys by staff from the Alaska Department of Fish and Game (ADF&G). Additional collection information, including sampling years and sample sizes, is found in Table 1. For this study, wild Unuk River samples (Unuk‐W) were used from collections in 1988 (Cripple Creek) and 2004 (Clear Creek), and Little Port Walter samples (Unuk‐H) were collected in 2018 (GT‐seq) and 2020 (lcWGS). Phenotypic data was collected for all Unuk‐H fish returning to LPW (the only population in this study with individual phenotypic data), including weight, length, sex, and age of return. Over the research hatchery program's duration, most individuals returned at age five (50%), followed by those of age four (25%), age six (14%), age three (9%), and a few fish at other ages. From these proportions, the average age of return was assumed to be five years; therefore, the number of generations of hatchery rearing since broodstock initiation ranges from at least five to no more than nine generations. The wide range is due to the infusion of wild gametes in 1998, but the true number of generations is likely closer to nine given the small incorporation of gametes.

Wild samples from the Chickamin River (Chickamin‐W) were collected in 1990 and 2005 (both South Fork), and the corresponding hatchery samples at Whitman Lake (Chickamin‐H) were collected in 2014. The majority of Chinook returning to Whitman Lake are age five (51.7%) or age four (34.9%), so approximately six to seven generations have passed since hatchery broodstock initiation (Tessa Frost (SSRAA), personal communication). Wild samples from Andrew Creek (Andrew‐W) were collected in 1989 and 2004, and the corresponding samples at Macaulay Hatchery (Andrew‐H) were collected in 2014. Since 58% of Andrew Creek Chinook return at age five (ages four and six each represent approximately 20% of returns), an estimated generation time of five years results in approximately eight generations of hatchery rearing since derivation from the progenitor stock (L. Wilson (ADF&G), personal communication).

### 2.3 Analysis of population structure, genetic diversity, and effective population size with GT‐seq data

DNA from all tissue samples was extracted with Qiagen DNeasy Blood and Tissue Extraction kits using the manufacturer's protocols (Hilden, Germany). A genotyping‐in‐thousands by sequencing (GT‐seq) dataset was generated to obtain high‐coverage genotypes for our study populations, which was used to assess population genetic metrics and compare them to estimates obtained with low‐coverage whole‐genome sequencing (lcWGS). GT‐seq genotyping was conducted following the methods of Campbell et al. (2015) to genotype a panel comprising 299 loci, which is frequently used for Chinook salmon management across their range (Barclay et al., 2019; Hess et al., 2015). The dataset was reduced to 254 loci following filtering of loci that were potentially paralogous, were out of Hardy–Weinberg expectations, or displayed significant pairwise linkage disequilibrium (see Shedd & Gilk‐Baumer, 2021 for details). Individuals were removed if missing genotypes >20% of loci or identified as duplicates sharing genotypes >95% of loci. Population structure was investigated by calculating pairwise *F*_ST_ (Weir & Cockerham, 1984) in Genepop v4 (Rousset, 2008), and patterns were visualized with a principal coordinate analysis (PCoA) constructed in GenAlEx v6.5 (Peakall & Smouse, 2012) using standardized distance‐based covariance. With less than 300 SNPs in the GT‐seq panel, a population‐level PCoA was used based on the distance matrix (*F*_ST_) rather than raw genotype likelihoods such as in a PCA (Peakall & Smouse, 2012). GenAlEx was also used to calculate observed and expected heterozygosities (*H*_O_ and *H*_E_). Effective population sizes (N_e_) were estimated using the linkage disequilibrium method implemented in NeEstimator v2.1 (Do et al., 2014), with a critical value set to 0.05 to remove rare alleles.

### 2.4 Whole‐genome sequencing library preparation

Whole‐genome library preparation followed the methods of Baym et al. (2015) and Therkildsen and Palumbi (2017), modified by Euclide et al. (2023). Briefly, input DNA was normalized to 10 ng for each individual, and libraries were purified and normalized using SequalPrep plates (ThermoFisher Scientific, Waltham, MA, USA). Normalized pooled libraries were subject to a 0.6× size selection, purification, and volume concentration with AMPure XP. Samples were sent to Novogene (Sacramento, CA) for whole‐genome sequencing using paired‐end 150‐bp reads on an Illumina NovaSeq S4. Ninety‐six individuals were multiplexed on a lane to target a genome‐wide depth of coverage of 3× per individual.

### 2.5 Sequence alignment and genotype likelihood estimation

Fastq reads were aligned to the Chinook salmon reference genome (Otsh_v1.0; GFA_002872995.1; Christensen et al., 2018) using BWA‐MEM v0.7.17 with default parameters (Li & Durbin, 2009). The aligned reads were processed with SAMtools v1.18 and converted to sorted bam files using default parameters. Individuals with a depth of coverage lower than 1× were removed. Then, ANGSD v0.930 (Korneliussen et al., 2014) was used to call SNPs, and genotype likelihoods were determined with the SAMtools model (GL 1) for 190 individuals. For each SNP call, the minimum minor allele frequency was set at 5% (minMaf 0.05), and a *p*‐value cutoff of 10^−10^ was used to remove rare alleles and low‐confidence SNPs (snp_pval 1e‐10). The minimum number of individuals with genotype likelihoods at a polymorphic locus was set to 70% of the total (minInd 133), the minimum depth of coverage was set to the total number of individuals (setminDepth 190), and the maximum depth was set to the total number of individuals multiplied by twice the coverage, which was set to four to account for individuals with greater coverage (setmaxDepth 1500). Genotype likelihoods with at least a 99% base call accuracy (minQ 20) and mapping accuracy (minMapQ 20) were retained. Major and minor alleles for all individuals were determined from genotype likelihoods (doMajorMinor 1).

### 2.6 Genome‐wide population genetic analysis

To explore genetic divergence across populations, principal component analyses (PCAs) were conducted using PCAngsd v1.10 (Meisner & Albrechtsen, 2018). Initially, wild samples were analyzed by year to investigate temporal structure. Temporal replicates clustered together in PCAs (data not shown), and wild samples from each site were therefore combined into single populations. To determine weighted pairwise *F*_ST_s (Weir and Cockerham's) for the three hatchery–wild pairs, site allele frequency likelihoods were calculated in ANGSD (doSaf 1) using the same filtering criteria as above for each population, except the SNP *p‐*value cutoff was set to 10^−6^. Global and genome‐wide *F*_ST_s were calculated using the folded site frequency spectrum for each hatchery–wild pair (realSFS). Heterozygosity was calculated across the genome with ngsTools' ngsStat (Fumagalli et al., 2013) and averaged to compare genetic diversity within and across populations.

### 2.7 Identification and characterization of regions with high genomic divergence

Manhattan plots of *F*_ST_ values for each SNP were plotted in R to visualize genetic differentiation across hatchery and wild pairs. We then used a local score approach (Fariello et al., 2017) to investigate genomic regions that may be responding to domestication selection. Local scores incorporate differentiation and linkage disequilibrium information for neighboring loci to identify genomic regions that are putatively under selection (Fariello et al., 2017). To calculate local scores, counts for each nucleotide were calculated separately for each of the six populations in ANGSD (doCounts 1, dumpCounts 3, skipTriallelic 1). Only loci that were found in both the hatchery and wild populations were retained, and Allele 1 and Allele 2 were standardized across populations. For each locus, a Fisher's Exact test for significant allele frequency differences was performed between the two populations, resulting in a *p*‐value at each SNP. The *p*‐values were used as input for the local score approach with a specified smoothing parameter (ξ) of 2. Significance thresholds were calculated for each chromosome (α = 0.01), and any regions that exceeded the threshold were determined to be outlier peaks.

We also used Bayescan v2.1 (Foll & Gaggiotti, 2008) to provide an additional and largely independent line of evidence for selection at outlier peaks identified by local score. Bayescan was conducted on all SNPs within chromosomes that contained an outlier peak. For all SNPs within the specified chromosome, minor allele frequencies for each population were converted to counts for the major and minor alleles (total number of alleles = double the sample size to represent diploidy). All parameters were set to the default, and a false discovery rate (FDR) corrected *q*‐value of 0.01 (−log(*q*‐value) = 2) was the outlier SNP cutoff. Any outlier peaks identified via the local score method that did not contain outlier SNPs in Bayescan were removed. Peak locations were compared across each hatchery–wild pair to determine if they were shared.

Additionally, genotype heatmaps were created for all outlier peaks in 10 kb regions, using the peaks' highest *F*_ST_ SNP as the midpoint, to further visualize allele frequency patterns in peaks. Genotype likelihoods were converted to genotype calls to simplify the heatmaps and provide contrast between individuals and populations.

In addition to the two outlier detection methods described above, we also calculated the absolute genetic divergence between populations (*D*_*xy*_) on a per‐SNP basis using ngsTools' *getD*_*xy*_.*pl* script (Fumagalli et al., 2014). *D*_*xy*_ measures how much one population's nucleotides deviate from another population's (Burri et al., 2015), in this case, the hatchery population from its progenitor wild population. It should be noted that *D*_*xy*_ is only calculated across variant sites using this program, which may limit the interpretability of *D*_*xy*_ results (Cruickshank & Hahn, 2014). First, *D*_*xy*_ was plotted across the entire genome. Then, to investigate patterns of *D*_*xy*_ within outlier peaks, *D*_*xy*_ was evaluated across adjacent windows of size 10 kilobases (kb). SNPs with *D*_*xy*_ above 0.5 were counted within each window to determine the density of high‐*D*_*xy*_ SNPs, and windows with densities in the top 1% of the retained SNPs were considered to have elevated *D*_*xy*_. In other words, windows were considered elevated if they ranked in the 99th percentile for the number of SNPs greater than 0.5. Only *D*_*xy*_ values greater than 0.5 were used because they represent SNPs that switched from the major allele in one population (wild) to the minor allele in the comparison population (hatchery), which could indicate either selection with gene flow or an ancient balanced polymorphism, represented as a *D*_*xy*_ peak (as opposed to background selection, which is expected to show a dip in *D*_*xy*_ values; Han et al., 2017).

Linkage disequilibrium (*r* ^2^) was calculated in ngsLD v1.1.1 (Fox et al., 2019) within each identified outlier peak for each hatchery–wild comparison. Within each peak, LD was calculated for all SNP pairs within 5 kb of either side of the peaks' highest *F*_ST_ SNP position (for a total of 10 kb). Heatmaps were used to visualize *r* ^2^ values, but they were mapped over a greater distance of 100 kb. After investigating the patterns of LD between hatchery and wild populations separately, population pairs were combined for the following LD analyses to increase the sample size.

Linkage disequilibrium in the peaks was tested against background rates of LD to determine if LD was elevated in peak regions. For each chromosome on which a peak was identified, ten regions were randomly selected from that chromosome to represent background LD levels (of size 10 kb to match maximum distance as LD in peaks); *r* ^2^ values were estimated in each of the ten random regions and then pooled together. Then, to compare background LD to LD in peaks, a randomization without replacement method was repeated 1000 times on these pooled values. To maintain equal sample sizes, the number of *r* ^2^ values in the pooled background regions was equal to the number of *r* ^2^ values in the corresponding outlier peak. For each randomization permutation, a one‐tailed Wilcoxon Rank‐Sum test (α = 0.05) was performed to compare the outlier peak *r* ^2^ values to the background *r* ^2^ values. If 95% of the permutations showed a significant elevation of *r* ^2^ values in the peak compared to the background, then the peak was determined to be significantly elevated. Violin plots were utilized to visualize differences between the distributions of *r* ^2^ values in peaks compared to the background region *r* ^2^ values. Finally, LD was calculated and compared against background regions using the same methods above for all locations in which a peak was found regardless of the population pairs (e.g., if Chr 1 contained a peak in the Unuk H/W comparison, LD would be tested in that same region across the Andrew H/W and Chickamin H/W population pairs). This was conducted to determine if patterns of LD were consistent across populations regardless of the identification of an outlier peak in that comparison and, therefore, if elevated LD was inherent to that region of the genome.

### 2.8 Functional significance of peaks

To investigate the functions of genes within outlier peaks, genes within peaks were compared with all Chinook salmon protein‐coding genes in a GO enrichment test. Briefly, protein‐coding Chinook salmon genes were downloaded from NCBI (https://www.ncbi.nlm.nih.gov/genome/13133?genome_assembly_id=360171), and these sequences were compared with the zebrafish (*Danio rerio*) protein database (RefSeq protein coding database) using BlastX with default parameters, except for a maximum e‐value of 1 × 10^−8^ and a maximum of 25 protein hits per gene (Camacho et al., 2009). GO terms associated with the zebrafish proteins were downloaded using Blast2GO. Then, GO terms associated with genes within peaks were compared to GO terms associated with protein‐coding genes not found in peaks using a Fisher's exact test (FDR‐corrected *p*‐value < 0.001).

## 3 RESULTS

### 3.1 Sequencing metrics

Low‐coverage whole‐genome sequencing (lcWGS) produced an average of 63 million reads across each of the 190 individuals in the lcWGS dataset. Two individuals with a depth of coverage less than 1× (0.06× and 0.07×) were removed from the Unuk‐H population. Across all individuals, the average percent coverage for each base pair was 78%, and the average depth of coverage was 3.6× (range = 1.4–6.8). After quality filtering and SNP scoring, the final set of retained genotype likelihoods for each population averaged 7.2 million SNPs (range = 6,053,281–8,511,491).

### 3.2 Population structure and genetic diversity

**FIGURE 2:**
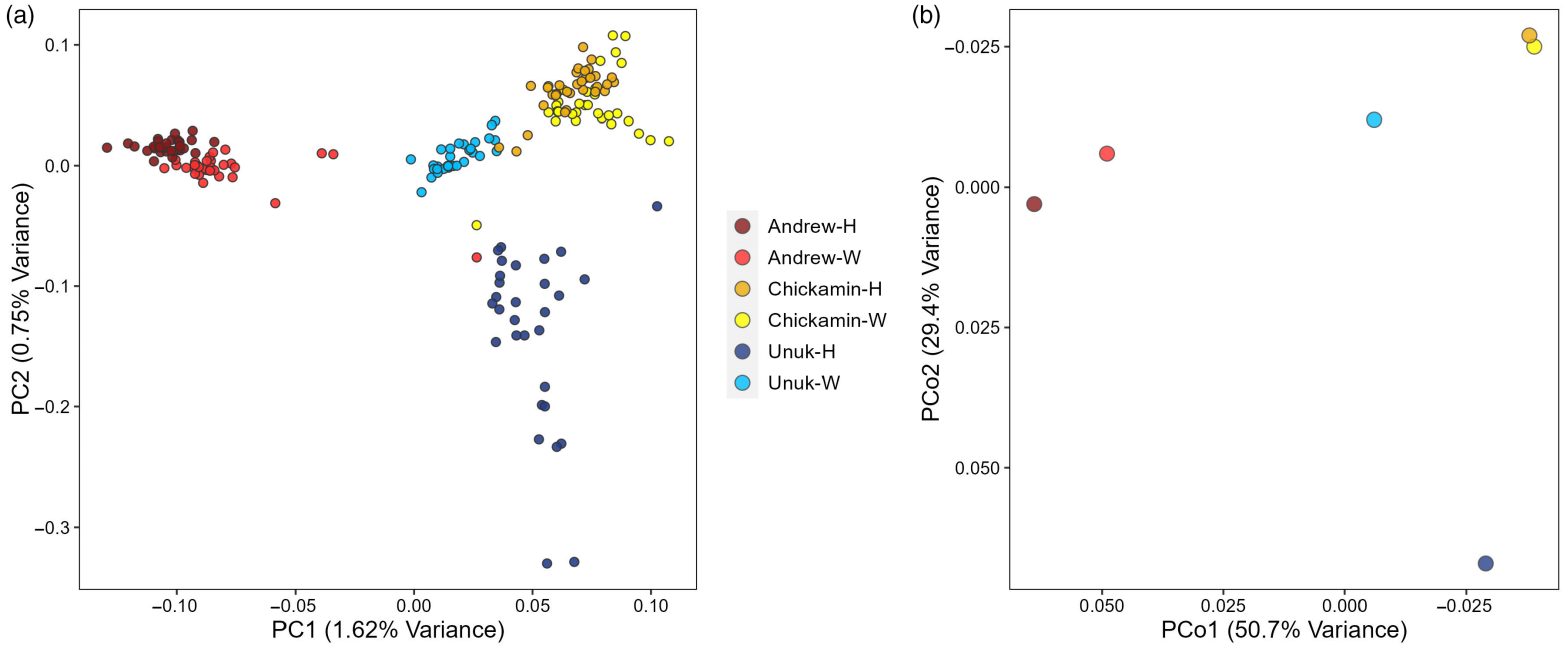
(a) Principal component analysis (PCA) from lcWGS data for each individual sample, and (b) principal coordinate analysis (PCoA) from GT‐sequencing data for each population.

Patterns of population structure were similar between the individual‐based PCA constructed from 1.1 million SNPs genotyped from lcWGS and the population‐based PCoA constructed from 254 SNPs genotyped with the genotyping‐in‐thousands by sequencing (GT‐seq) panel (Figure 2). Wild populations were largely differentiated across PC1, which accounted for 1.62% of the variation (Figure 2a). Unuk‐W and Chickamin‐W were adjacent in the PCA, which is reflective of their geographic proximity (see Figure 1). Samples from Chickamin‐H and Chickamin‐W were the most genetically similar of all hatchery–wild pairs, followed by Andrew‐H and Andrew‐W, which showed overlap across individuals in the PCA. Unuk‐H was the most dispersed population and did not overlap with Unuk‐W, suggesting a higher degree of genetic differentiation between Unuk‐H and Unuk‐W relative to the other population comparisons. Furthermore, Unuk‐H had the most within‐population variability. One individual from Chickamin‐W and another from Andrew‐W clustered near Unuk‐H, which was not explained by data quality (both had depth of coverage greater than four, suggesting this was not due to poor sequencing) but could possibly be due to straying or introgression of hatchery individuals with wild populations.

Estimates of N_e_ were variable and ranged from 92 in the Unuk‐H population to 1582 in the Andrew‐W population (Table 1). Effective sizes in hatchery populations were consistently lower than their wild founding populations, and this was most prevalent in the Unuk line, where the *N*_e_ of Unuk‐W was 15 times that of Unuk‐H. The Andrew‐W population had an *N*_e_ approximately three times greater than Andrew‐H, while the *N*_e_ in the Chickamin‐W population was about one‐third higher than the hatchery line. Observed heterozygosity from lcWGS and GT‐seq was similar but slightly lower for GT‐seq estimates compared to lcWGS (Table 1). Observed and expected heterozygosities calculated with GT‐seq were similar in all populations, suggesting that there were no substantial reductions in genetic diversity according to these metrics despite the lower effective sizes of the hatchery populations.

Global *F*_ST_ estimates between all populations showed similar patterns as the PCA and PCoA and revealed low‐to‐moderate genetic differentiation among populations. The GT‐seq estimates were generally analogous to lcWGS estimates (average *F*_ST_ with GT‐seq = 0.018, average *F*_ST_ with lcWGS = 0.016; Table 2), and *F*_ST_ estimates were greatest between Andrew‐H and Unuk‐H for both methods (GT‐seq *F*_ST_ = 0.0291; lcWGS *F*_ST_ = 0.0231). Of the three hatchery–wild pairs, Andrew H/W had the lowest combined estimates (GT‐seq *F*_ST_ = 0.0033; lcWGS *F*_ST_ = 0.0088), whereas Unuk H/W had the greatest differentiation (GT‐seq *F*_ST_ = 0.0184; lcWGS *F*_ST_ = 0.0138), which aligns with visualized genetic distances from the PCA and PCoA as well as differences in *N*_e_ across hatchery populations.

**TABLE 2:** Pairwise global *F*_ST_s across all loci with lcWGS (below the diagonal) and GT‐seq (above the diagonal).

	Unuk‐H	Unuk‐W	Andrew‐H	Andrew‐W	Chickamin‐H	Chickamin‐W
Unuk‐H	**0**	**0.0184**	0.0291	0.0263	0.0220	0.0211
Unuk‐W	**0.0138**	**0**	0.0162	0.0117	0.0118	0.0080
Andrew‐H	0.0231	0.0162	**0**	**0.0033**	0.0284	0.0268
Andrew‐W	0.0194	0.0137	**0.0088**	**0**	0.0202	0.0217
Chickamin‐H	0.0182	0.0113	0.0222	0.0204	**0**	**0.0079**
Chickamin‐W	0.0172	0.0110	0.0218	0.0193	**0.0103**	**0**

*Note*: Darker red colors represent greater *F*_ST_ values. Bold values are hatchery–wild population pair comparisons.

### 3.3 Identification and characterization of regions with high genomic divergence

**FIGURE 3:**
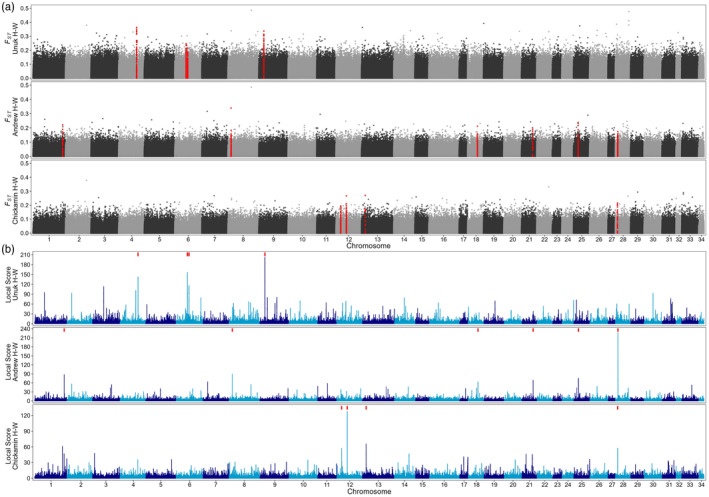
Genome‐wide Manhattan plots displaying (a) *F*_ST_ and (b) local score for each of the three hatchery–wild population pairs: Andrew‐H/W, Unuk‐H/W, and Chickamin‐H/W. Alternating grays (*F*_ST_) and blues (local score) represent different chromosomes. Red points in (a) are loci within identified outlier peaks, and red boxes at the top of the y‐axis in (b) represent locations of outlier peaks.

We identified a total of 14 outlier peaks between hatchery–wild pairs using the local score approach: four in Unuk, six in Andrew, and four in Chickamin (Figure 3; Table 3; see Table S1 for chromosomal significance thresholds and Table S2 for peak‐specific results). Using the boundaries defined from the local score method, the average peak size was 35.4 kb (range = 1.5 to 85 kb). Peaks were largest in the Unuk comparison (90.9 kb on average), followed by Andrew (45.2 kb average) and Chickamin (26.7 kb average; Table 3). All peaks contained at least one Bayescan outlier SNP with an average of ten outliers per peak (range = 2–30), and the smallest *q*‐value for over half the peaks was zero (Table S2; Suppl. Peak Results). Therefore, all local score peaks were retained because they were supported by two outlier detection methods.

**TABLE 3:** Number of outlier peaks per comparison and additional descriptions of the peaks, including average *F*_ST_, average size, total number of Bayescan outlier loci (*q*‐value >0.01) within all peaks and the corresponding range of total Bayescan outlier loci within each individual peak, total number of peaks with significantly greater LD (r^2^) and elevated *D*_*xy*_ compared to background values in each comparison, and the number of genes located within the peaks' boundaries for each comparison.

Pair	Stock	Number of peaks	Average *F*_ST_ in peaks	Average size of peaks (kb)	Bayescan outlier SNPs (n)	Significantly elevated LD	Elevated *D*_*xy*_	Number of genes
1	Unuk	4	0.081	90.9	73 (7–30)	3	4	12
2	Andrew	6	0.052	45.2	41 (3–15)	6	2	7
3	Chickamin	4	0.079	26.7	31 (2–22)	3	2	4

**FIGURE 4:**
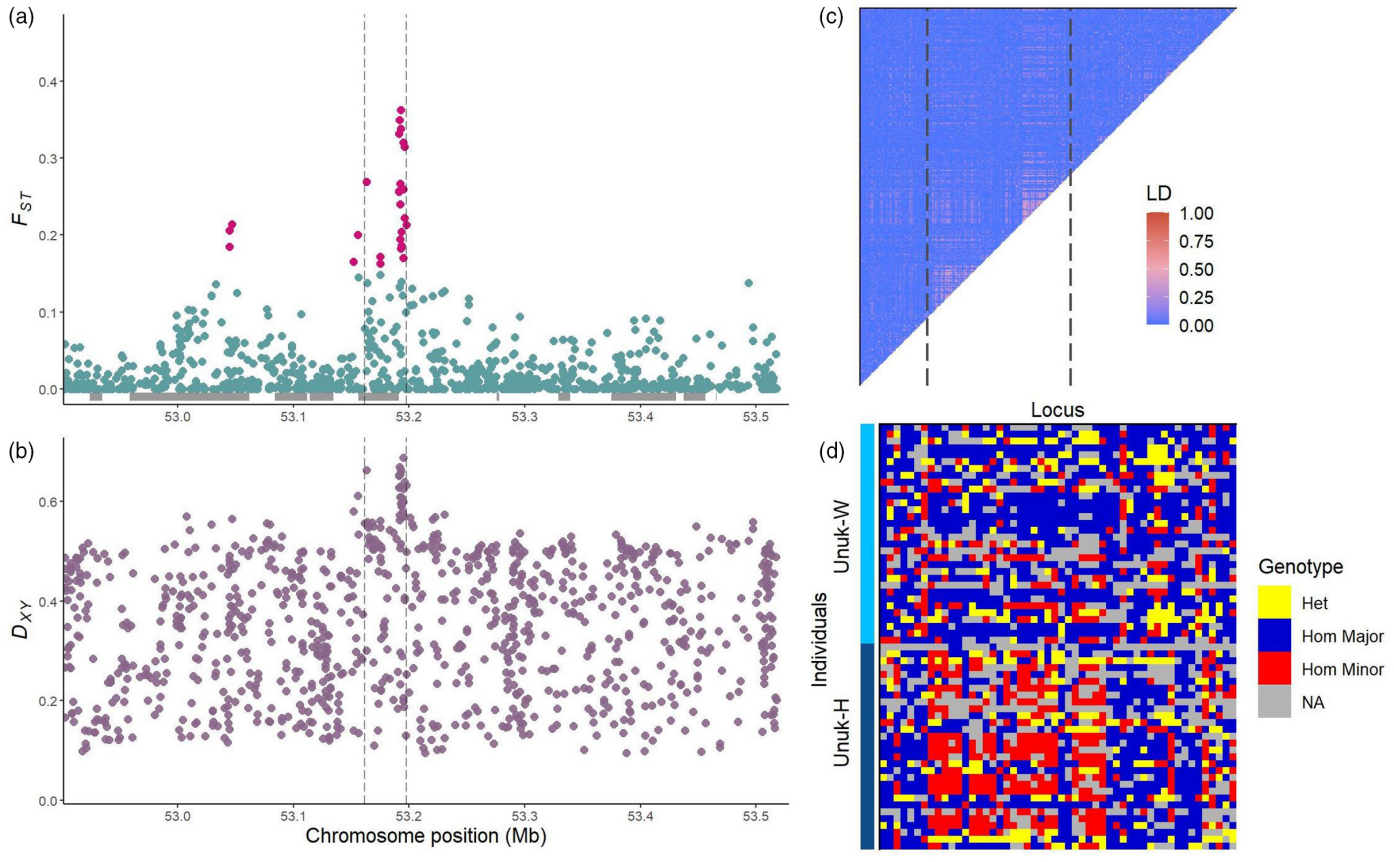
Unuk H/W on Chr 4 outlier peak around 53.2 Mb. (a) Manhattan plot of *F*_ST_ at each SNP in the vicinity of the outlier peak. Location of genes in this region is depicted as gray boxes directly above the x‐axis; (b) Manhattan plot of *D*_*xy*_; (c) LD (r^2^) heatmap of SNPs in the peak for hatchery and wild populations across a total of 100 kb; (d) Genotype heatmaps of SNPs across 10 kb for both hatchery and wild individuals in the Unuk comparison. Gray vertical dashed lines in A–C represent the outlier peak boundary as defined by the local score method.

**FIGURE 5:**
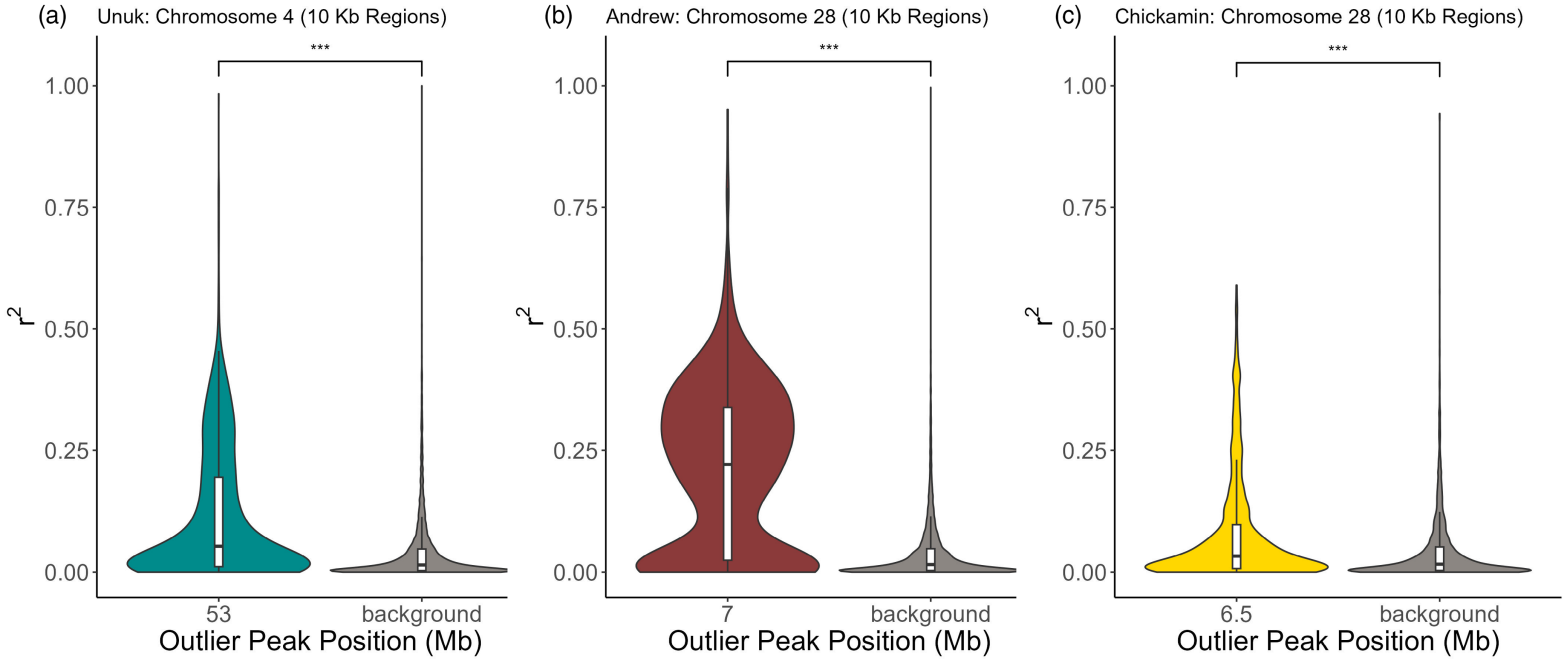
Violin plots of LD (r^2^) calculated across 10 kb regions for population pairs at three of the 14 outlier peaks in (a) Unuk on Chr 4 at 53.2 Mb, (b) Andrew on Chr 28 at 7.0 Mb, and (c) Chickamin on Chr 28 at 6.5 Mb. Corresponding boxplots are depicted as the white boxes inside the violin, where the white box displays the interquartile range and the black horizontal bar is the median. Outlier peaks were statistically compared to background LD on the same chromosome for each peak. ***p‐value < 0.0001.

None of the peaks were located in the same genomic region across the hatchery–wild pairs. The peaks that were closest to one another were on Chr 28, where the Chickamin peak was 800 kb downstream of the peak in the Andrew comparison (see Table S2 for peak positions). The most divergent peaks were found in the Unuk comparison, including a peak on Chr 4 with a maximum *F*_ST_ of 0.36 compared to an overall background *F*_ST_ of 0.01 (Figure 4a). Over half of the peaks (57%) also showed elevated absolute genetic divergence (*D*_*xy*_) (Table 3, Figure 4b), which is indicative of numerous major alleles in the peaks switching to minor alleles after a few generations in the hatchery environment as visualized in the gentoype heatmaps (Figure 4d). Additionally, elevated LD was documented in 86% of peaks (Table 3, Figures 4c and 5, Table S2).

Two peaks in the Unuk hatchery–wild comparison on chromosomes four and nine were particularly pronounced (Chr 4: Average *F*_ST_ = 0.094, Max *F*_ST_ = 0.363; Chr 9: Average *F*_ST_ = 0.112, Max *F*_ST_ = 0.337) compared to background *F*_ST_. Since the peak on Chr 4 has the greatest *F*_ST_ and high density of outlier SNPs, it was used as an example for peak‐specific analyses (Figure 4), although analyses were conducted on all peaks (see Suppl. Peak Results). The peak on Chr 4 is approximately 35.7 kb wide, and there are four protein‐coding genes within 100 kb of the max *F*_ST_ SNP (Figure 4a). The major allele at the highest *F*_ST_ SNP in this peak had a frequency of 0.91 in the wild population compared to 0.31 in the hatchery population, indicating a switch in the major allele between the hatchery and wild populations (Figure 4b). LD is also elevated in the peak (Figure 5a), and there is a low recombination region directly in the peak region (Figure 4c). This region of low recombination is potentially responsible for the haplotype blocks visible in the genotype heatmaps, where the predominant haplotype block is generally homozygous for the major allele in wild individuals and homozygous for the minor allele in hatchery individuals (Figure 4d). The heterozygous genotype also seems to be more prevalent in the hatchery population, which is likely a function of the more intermediate allele frequency in this population (Figure 4d). It is important to note that genotype heatmaps do not account for uncertainty from the original genotype likelihood data, which was removed to clearly elucidate haplotype patterns.

### 3.4 Functional significance of peaks

There were 23 genes located within outlier peaks (Tables 3 and S3). The two peaks with the greatest differentiation in the Unuk hatchery–wild comparison on Chr 4 and Chr 9 contained genes that code for contactin‐associated protein‐like, DNA damage‐binding protein, transmembrane protein 138, and CD151 antigen (Table S3). Biological processes associated with these proteins include structure formation during embryonic development, DNA repair and apoptotic cell processes, cilium assembly, and cell migration (Table S3). Although there were numerous genes in peaks, GO enrichment analysis did not find a single enriched GO term associated with these peaks. This suggests that the functions of protein‐coding genes within peaks are varied.

## 4 DISCUSSION

Domestication selection in salmon hatcheries can have a direct effect on fitness (Blouin et al., 2021; Christie et al., 2014; O'Sullivan et al., 2020), but little is known about the causative genetic mechanisms involved in these fitness reductions and whether molecular pathways are conserved between hatchery stocks (Gavery et al., 2018; Le Luyer et al., 2017; Mäkinen et al., 2015). We investigated this question using a combined approach of low‐coverage whole‐genome sequencing and genotyping‐in‐thousands by sequencing for three independent hatchery lines of Chinook salmon that were isolated from their wild progenitor stocks for five to nine generations. Hatchery lines were subtly to moderately diverged from their wild progenitor stocks and had lower effective population sizes but similar overall levels of genetic diversity. Outlier peaks with high differentiation between hatchery and progenitor stocks were relatively small (53 kb on average) and often showed signals of elevated linkage disequilibrium (LD) and absolute divergence (*D*_*xy*_). We did not identify shared peaks among hatchery–wild pairwise comparisons, suggesting that the genetic architecture of domestication selection varies between hatchery populations. Our study directly compares independent hatchery–wild population pairs, and the results provide fine‐scale genomic evidence of domestication selection in Pacific salmon, which adds to the growing body of research on salmon domestication and has potential implications for hatchery management and conservation.

### 4.1 Population divergence and diversity

Population structure in the wild samples reflected geography, where populations were separated by drainage, and the proximate Unuk and Chickamin River populations grouped more closely to each other than to Andrew Creek. This pattern of isolation by distance and population structure partitioned by drainage and life history is typical for Chinook salmon (e.g., Moran et al., 2013; Shedd & Gilk‐Baumer, 2021; Templin et al., 2011). The Andrew Creek and Chickamin hatchery lines were more genetically similar to their progenitor stocks than the Unuk hatchery stock. In fact, Unuk H/W *F*_ST_ was similar to or exceeded that of Unuk‐W compared to the other two wild populations, demonstrating that hatchery populations with small N_e_ can genetically diverge from their progenitor stocks after as few as five generations (c.f. Eldridge et al., 2009; Waters et al., 2015).

The rapid divergence between Unuk‐W and Unuk‐H is likely due to the low effective population size (*N*_e_ = 92) in Unuk‐H, which is lower than the *N*_e_ estimates for the Andrew‐H and Chickamin‐H hatcheries (535 and 264, respectively). The Unuk‐H population also displayed the greatest reduction in *N*_e_ compared to its progenitor stock (over an order of magnitude), whereas reductions in *N*_e_ in the other two populations were less pronounced. This reduction in *N*_e_ was expected because Unuk‐H is a small, research‐focused hatchery, while Andrew‐H and Chickamin‐H are propagated at larger production‐focused hatcheries. As a result, the LPW Research Station (Unuk‐H) uses a smaller number of individuals for broodstock than the two production‐focused hatcheries (average broodstock = 98 for Unuk‐H over the last ten years compared to 864 and 430 for Chickamin‐H and Andrew‐H, respectively; L. Wilson (ADF&G), personal communication). Another potential reason for low *N*_e_ in the Unuk‐H population is that it is a closed population without gene flow with other stocks due to 100% coded wire tagging and real‐time broodstock screening prior to spawning. In contrast, only 10%–15% of the other two hatchery stocks are tagged (RMIS, 1977), there is no broodstock screening, and gametes of the same stock are occasionally exchanged between hatchery facilities. Interestingly, we did not document a consistent reduction in heterozygosity in hatchery populations despite the lower effective population sizes; however, heterozygosity is a relatively insensitive indicator of population bottlenecks since it decreases at a slower rate (Allendorf, 1986).

### 4.2 Characteristics of outlier peaks

Of the 14 outlier peaks identified in this study, the majority are in regions of elevated *D*_*xy*_ (57%) and elevated LD (86%) compared to background regions of the genome. Thus, it is likely that many of the peaks have occurred due to directional selection on standing genetic variation present in the founding wild populations (Cruickshank & Hahn, 2014; Han et al., 2017). However, support for this interpretation comes from previous studies on highly diverged species and/or systems where divergence with gene flow is ongoing (Burri et al., 2015; Han et al., 2017). Nevertheless, selection is still expected to be the predominant force of genetic divergence in the early stages of a population split (Buffalo & Coop, 2020; Delmore et al., 2018; Renaut et al., 2013; Stankowski et al., 2019), even when gene flow is low (Chase et al., 2021), such as in this study. This study therefore indicates that the patterns of elevated *D*_*xy*_ and low recombination in divergent regions of the genome (i.e., outlier peaks) may also be due to selection when both divergence times and gene flow between hatchery and wild populations are low.

It is also important to note that although we found evidence of selection in peaks associated with high LD, this is likely, in part, due to the increased ease of detection rather than a requirement for selection to occur. In other words, local score methodologies leverage information on LD to build cumulative evidence for selection based on multiple loci in a given genomic region (Fariello et al., 2017) and may, therefore, fail to detect isolated loci with lower LD that may still be involved in local adaptations. Many highly differentiated *F*_ST_ SNPs were not in peaks, yet they were identified as potential targets of selection according to the results from Bayescan. It is certainly possible that those SNPs were a product of domestication selection and contributed to the polygenic nature of adaptation to the hatchery environment.

The identified outlier peaks are relatively narrow (3–158 kb) compared to those identified in many other studies of local adaptation (Clucas et al., 2019; Duranton et al., 2018; Ford et al., 2023; Thompson et al., 2020). This is especially true when compared to *F*_ST_ peaks found in systems with high gene flow, where blocks of high differentiation and low recombination often span megabases (Clucas et al., 2019; Huang et al., 2020; Via, 2012). Theory and empirical evidence suggest that outlier peaks should be narrower in lower gene flow systems because gene flow is not acting to disrupt co‐adapted alleles (Shi et al., 2023; Yeaman & Whitlock, 2011). However, wide peaks due to structural variants (SVs) can still facilitate rapid adaptation in the absence of gene flow (Therkildsen et al., 2019). Additionally, Bertolotti et al. (2020) documented differentiating SVs between domesticated and wild Atlantic salmon, but they were mainly smaller SVs comprising a few thousand nucleotides. However, we did not detect any evidence for SVs in the outlier peaks.

None of the outlier peaks identified were shared across hatchery–wild pairs, suggesting that adaptation to the hatchery environment is unique to each population. The only region that showed potential for parallel differentiation in outlier peaks was on Chr 28, where peaks identified in the Chickamin and Andrew lines were 800 kb apart. However, there were no enriched GO terms in the identified peaks, suggesting that genes found in differentiating regions are involved in unrelated processes.

Although we hypothesize that selection is the main force involved in creating the outlier peaks we identified, it is possible that outlier peaks may be confounded by the stochastic effects of drift, especially in the Unuk‐H population due to its lower *N*_e_ (Biswas & Akey, 2006). Many previous studies have demonstrated that false positive rates for single locus outlier tests can be high, especially when genetic differentiation is also high (reviewed in Hoban et al., 2016). However, we feel our conclusions are substantially strengthened by the local score approach, which is known to be robust in detecting selection across a gradient of genetic drift scenarios (Fariello et al., 2017). However, we cannot definitively conclude that the peaks identified are under selection. Some potential directions for future studies to further evaluate our conclusions include demographic modeling or designing a high‐throughput approach to screen a large number of individuals in outlier regions.

### 4.3 Comparison to previous domestication selection studies

Our findings are consistent with previous studies investigating the genetic basis of domestication selection in multiple aquaculture lines of Atlantic salmon, which also found few signals of parallel regions of divergence (López et al., 2019; Mäkinen et al., 2015). Few genomic studies in Pacific salmon have evaluated differentiation between multiple hatchery–wild population pairs, yet our results can be compared to those that investigated domestication in individual Chinook salmon hatcheries. Waters et al. (2015, 2018) investigated signatures of domestication selection in Chinook salmon and identified genomic regions where loci associated with fitness‐related traits (i.e., weight and run timing) overlapped with regions of adaptive divergence between the hatchery and wild populations. However, they also noted that they likely did not identify all differentiating genomic regions due to the lower density of markers generated through RAD sequencing (thousands of loci rather than millions with lcWGS; Waters et al., 2018). Recently, Ford et al. (2023) used whole‐genome sequencing to investigate domestication in spring‐run Chinook salmon from the Upper Columbia River and documented a peak of divergence between hatchery and wild populations on Ots08. Interestingly, this peak was near a locus associated with spawn timing documented in Waters et al. (2018). This peak was also one megabase (Mb) downstream of the peak found in the Andrew H/W comparison, suggesting that the genetic basis of domestication may be at least partially conserved. Nonetheless, the overwhelming evidence from our study and previous studies suggests that domestication selection generally involves changes across many genomic regions.

The growing body of research on domestication selection strongly suggests that adaptation to the hatchery environment is occurring through both direct and indirect genomic changes (Koch et al., 2023). Multiple studies have documented substantial differences in methylation and gene expression patterns between hatchery and wild populations that were shared across multiple hatchery lines (Koch et al., 2023). Although some studies suggest this may be the predominant mechanism (Gavery et al., 2018), multiple genomic mechanisms may be interacting to influence hatchery domestication. Furthermore, there is potential for the inheritance of epigenomic variation in hatchery salmon (Gavery et al., 2018; Leitwein et al., 2021), yet the heritability of such variation is likely lower than direct genomic changes. If hatchery practices are modified to reduce domestication, reversal of genomic changes may be more difficult than epigenetic changes. Thus, future research should focus on understanding their relative influences and investigating whether specific hatchery practices may be able to slow or reverse both direct and indirect genetic changes caused by hatchery rearing.

### 4.4 Possible mechanisms for variation between hatchery–wild population pairs: Genomic architecture of parallel adaptation and differing hatchery practices

Research on domestication in other species indicates that adaptation to captivity involves traits and behaviors that are highly polygenic (Carneiro et al., 2014; Stetter et al., 2018). Both empirical and simulation‐based studies suggest that polygenic traits also tend to be highly redundant (i.e., multiple genotypes can give rise to the same phenotype; Láruson et al., 2020), allowing for unique genetic architectures to underlie adaptation (Barghi et al., 2019, 2020; Yeaman, 2015). More specifically, parallel adaptation to similar environments has been shown to occur via variable genomic architectures (Ament‐Velásquez et al., 2022; Barghi et al., 2019; Bolnick et al., 2018; Schlötterer, 2023; Therkildsen et al., 2019). For example, ten *Drosophila* lines exposed to the same selective pressure evolved similar phenotypes but had unique genetic architectures underlying adaptation (Barghi et al., 2019). Our study may lend further support to these findings by identifying idiosyncratic genomic changes in response to domestication selection, suggesting that adaptation to captivity is likely polygenic.

It is possible that variable hatchery practices also played a role in the observed differences among hatchery–wild pairs, particularly the more pronounced signals of domestication selection in the Unuk‐H line. Theoretically, selection should be more effective at increasing the frequency of advantageous alleles in larger populations, such as those found in the Andrew‐H and Chickamin‐H lines (Lanfear et al., 2014); however, the most prominent *F*_ST_ peaks were found in the Unuk‐H line. This suggests that the selection coefficients responsible for domestication selection may be considerably stronger in the Unuk‐H line. Phenotypic changes attributed to domestication selection, most notably in maturation timing, have been previously documented in the Unuk‐H line, with hatchery fish maturing earlier than wild fish (Joyce et al., 2004). Typically, Little Port Walter collected Unuk‐H broodstock from early June to early August; the fish were then held in saltwater net pens with a one‐meter freshwater lens until gamete collection and spawning, which occurred across three weeks in August. Saltwater net pens are used at LPW to reduce bacterial kidney disease exposure in female Chinook, but may also negatively affect gamete quality, possibly resulting in differential family‐based survivals. In contrast, broodstock for Andrew‐H and Chickamin‐H are held exclusively in freshwater raceways for shorter durations. While the mechanisms are unclear, the difference in broodstock collection and holding practices at LPW may contribute to the variation in our results.

Another possible difference between Unuk and the other two population pairs is the average body size at release between the hatcheries. Size at release has been positively associated with smolt‐to‐adult survival (James et al., 2023), including Unuk‐H fish at LPW (Martin & Wertheimer, 1989). The average size at release for Unuk‐H smolts was five to seven grams larger than Andrew‐H and Chickamin‐H smolts (32.3 g, 26.7 g, and 25.5 g for Unuk‐H, Andrew‐H, and Chickamin‐H, respectively; RMIS, 1977). However, size at release for Unuk‐H smolts was also much more variable than the other two stocks (SD of 26.6 g, 6.5 g, and 4.4 g for Unuk‐H, Andrew‐H, and Chickamin‐H, respectively; RMIS, 1977), likely due to experiments at LPW that deliberately manipulated average size at release. The combination of larger but more variable release sizes for Unuk‐H fish may have exacerbated differences in family‐based survival and thus selection coefficients, as growth and mass at smoltification in salmonids have a genetic basis (Carlson & Seamons, 2008).

Environmental differences between wild populations and the respective hatchery locations may also contribute to our results. Specifically, all three wild populations, along with the Andrew‐H and Chickamin‐H stocks, are located on the mainland. In contrast, the Unuk‐H stock is reared in a facility on Baranof Island that experiences a different thermal regime and substantially more precipitation than the other locations. Such environmental differences between the mainland and island rearing locations could have led to greater divergence between the Unuk‐W and Unuk‐H stocks. Other hatchery practices, such as mating designs, feeding regimes, rearing densities, and real‐time screening of broodstock (i.e., gene flow), may also explain the observed differences. While none of these differences in rearing practices presents an obvious explanation for the apparent differences in selection pressures, this study suggests that variation in hatchery practices may lead to variation in selection coefficients. The disproportionate genetic divergence across hatcheries illustrates the importance of monitoring hatchery lines for genetic and phenotypic signals of domestication selection, which may help determine which hatchery practices result in increased divergence. One approach to addressing variation in domestication selection due to cryptic differences in hatchery practices could be to compare signals of domestication selection in individuals derived from the same hatchery lineage but reared in different hatcheries.

### 4.5 Future directions and conclusions

Hatchery supplementation of wild populations is an increasingly relied‐upon management tool to support declining salmon fisheries (Paquet et al., 2011). However, our study and previous research have made it clear that domestication selection in hatcheries affects salmon genomes and epigenomes (Koch et al., 2023). Although it could greatly benefit hatchery management to implement genetic tools, detecting signals of domestication selection may be difficult if they are largely polygenic and population specific. Specifically, our study highlights the fact that hatcheries with generally comparable environmental conditions (i.e., segregated hatcheries in SEAK) can have varying genetic responses to domestication selection.

Fortunately, the genomic revolution has made it feasible to generate and screen genome‐wide data for associations connected with selection and the development of maladaptive phenotypes. Understanding the correlation between genomic polymorphisms and fitness‐related traits is critical to discovering information that could be useful for hatchery management. Our study, along with the body of research on this topic, highlights the importance of monitoring genetic and epigenetic divergence between hatchery and wild stocks. Studies that measure phenotypes (e.g., Waters et al., 2018) combined with high‐resolution genomic and epigenomic data have great potential to provide useful inferences that could ultimately be incorporated into hatchery management to further minimize domestication selection and protect wild stocks.

## CONFLICT OF INTEREST STATEMENT

The authors declare there are no competing interests.

## Supporting information


**Table S1.** Local score significance thresholds for each chromosome within each population comparison (α = 0.01; ξ = 2).
**Table S2**. Peak‐specific results, including the chromosomal position, size, average and maximum *F*_ST_ within each peak, and allele frequency for the maximum *F*_ST_ SNP in both the wild and hatchery populations. Bayescan outliers within the local score peak boundaries, including the number of outlier loci and the maximum −log(*q*‐value) in each peak. The average *p*‐value and significance (percent of permutations that were significant) and which other population pairs in the region showed elevated LD. Lastly, if *D*_*xy*_ in the peak region was within the 99th percentile (top 1%).
**Table S3**. Genes identified in outlier Fs peak, including the population each peak was found in, the chromosome, peak location, and start and end points of the gene. The corresponding gene name, protein name, and associated GO terms from Blast against the zebrafish genome. Additionally, other salmon domestication studies for which that same or paralogous gene was found.

**Data S1.** Supplemental Peak Results Document.

## Data Availability

Data for this study are available at NCBI's SRA database under the project ID PRJNA1069051.
