# Supplementary material for: Genomic evidence for domestication selection in three hatchery populations of Chinook salmon, Oncorhynchus tshawytscha
Source: Evol Appl. 2024 Feb 14;17(2):e13656. doi: 10.1111/eva.13656 (PMC10866082; doi:10.1111/eva.13656)
Supplement: Supplementary file 2 — Data S1. Supplemental Peak Results Document. [file EVA-17-e13656-s002.pdf]

Supplemental Peak Results Document

Population and chromosome is identified through the title of the figures.

Only chromosomes for which a peak was identified via the local score method are included.

Refer to Figure 4 (local F_{ST}, D_{xy}, LD heatmap, genotype heatmap) and Figure 5 (violin plots) in the main document for the peak specific analyses captions.

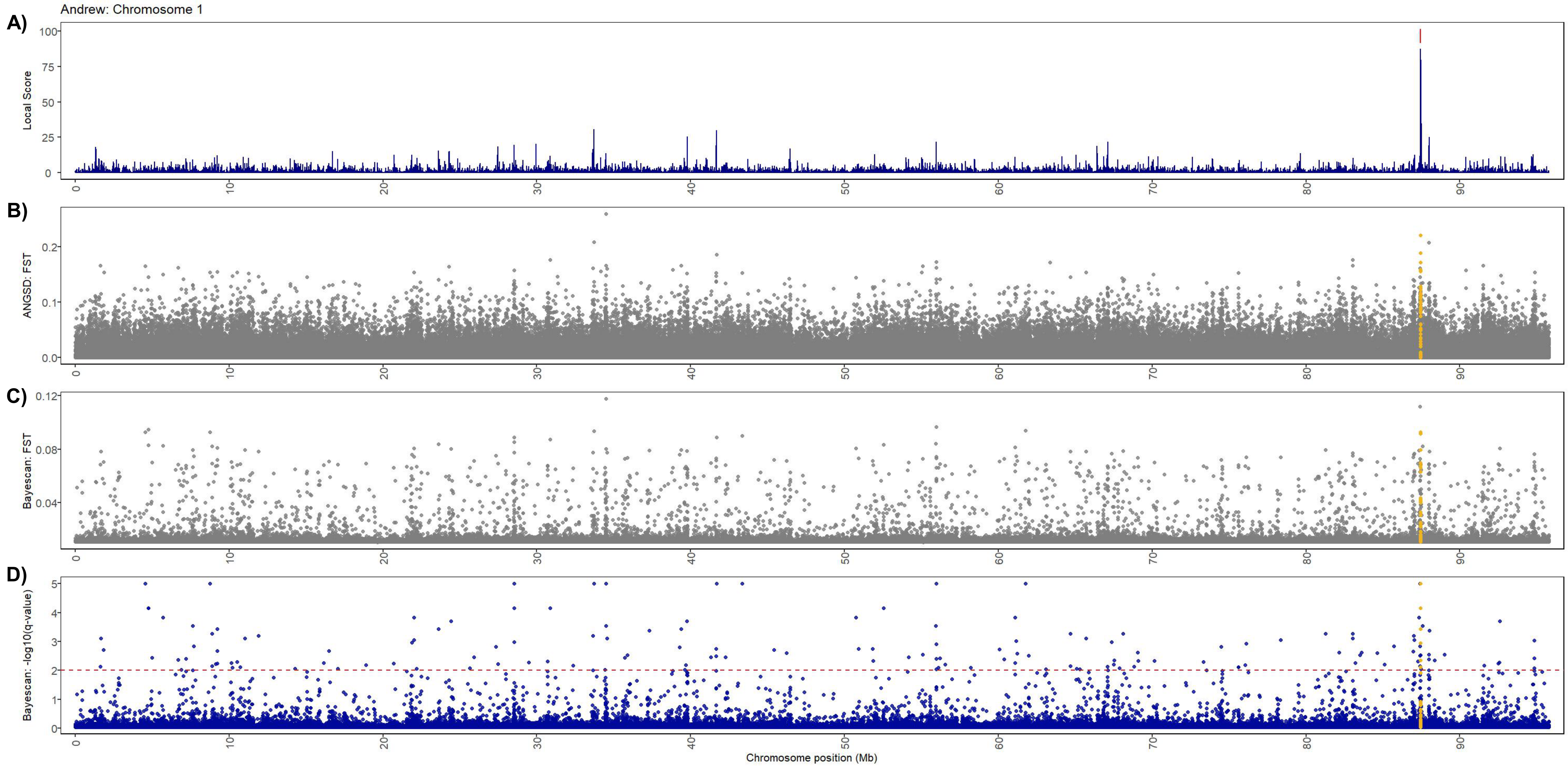

Manhattan plots for Andrew H-W comparison on chromosome 1. A) Local score plot with the red rectangle signifying an outlier peak identified through local score; B) FST calculated in ANGSD; C) FST calculated in Bayescan; D) –log(q-value) calculated in Bayescan with the red dashed line signifying the cutoff for outlier loci expected to be under selection. The yellow points in panels B – D are loci within the local score outlier peak boundaries.

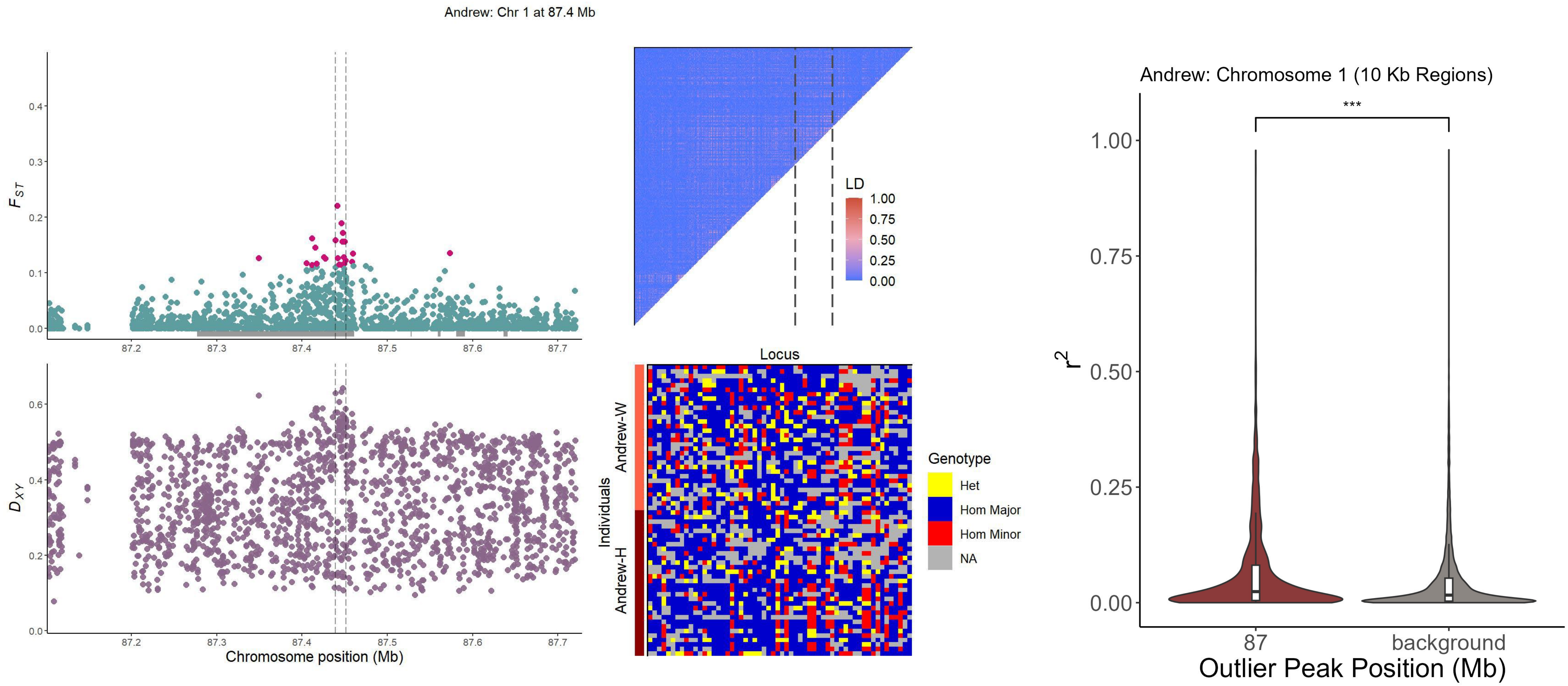
Andrew: Chr 1 at 87.4 Mb
F_{ST}
0.4
0.3
0.2
0.1
0.0
87.2
87.3
87.4
87.5
87.6
87.7
D_{XY}
0.6
0.4
0.2
0.0
Chromosome position (Mb)
LD
1.00
0.75
0.50
0.25
0.00
Locus
Individuals
Andrew-W
Andrew-H
Genotype
Het
Hom Major
Hom Minor
NA
Andrew: Chromosome 1 (10 Kb Regions)
***
r^2
1.00
0.75
0.50
0.25
0.00
87
background
Outlier Peak Position (Mb)

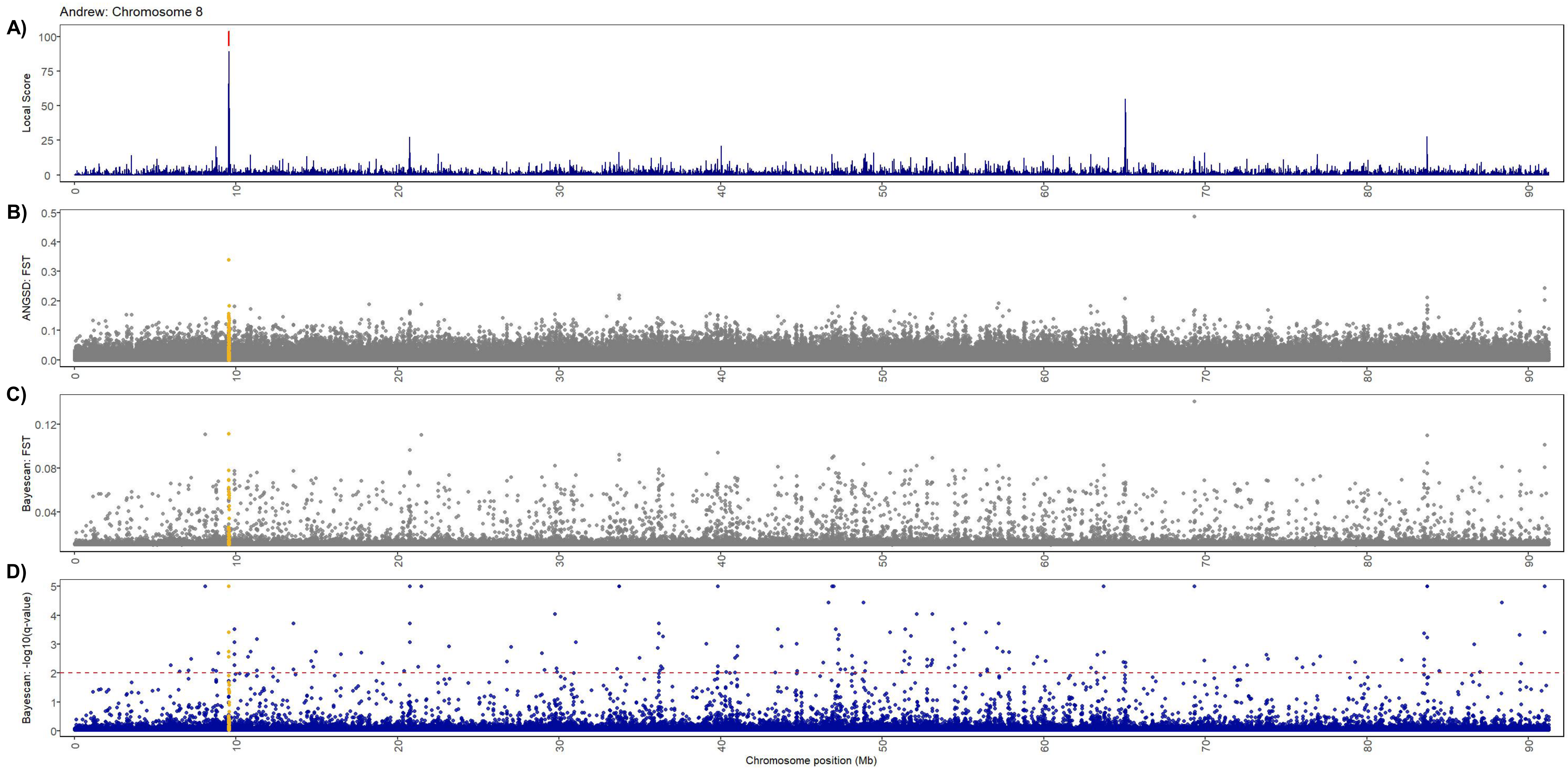

Manhattan plots for Andrew H-W comparison on chromosome 8. A) Local score plot with the red rectangle signifying an outlier peak identified through local score; B) FST calculated in ANGSD; C) FST calculated in Bayescan; D) –log(q-value) calculated in Bayescan with the red dashed line signifying the cutoff for outlier loci expected to be under selection. The yellow points in panels B – D are loci within the local score outlier peak boundaries.

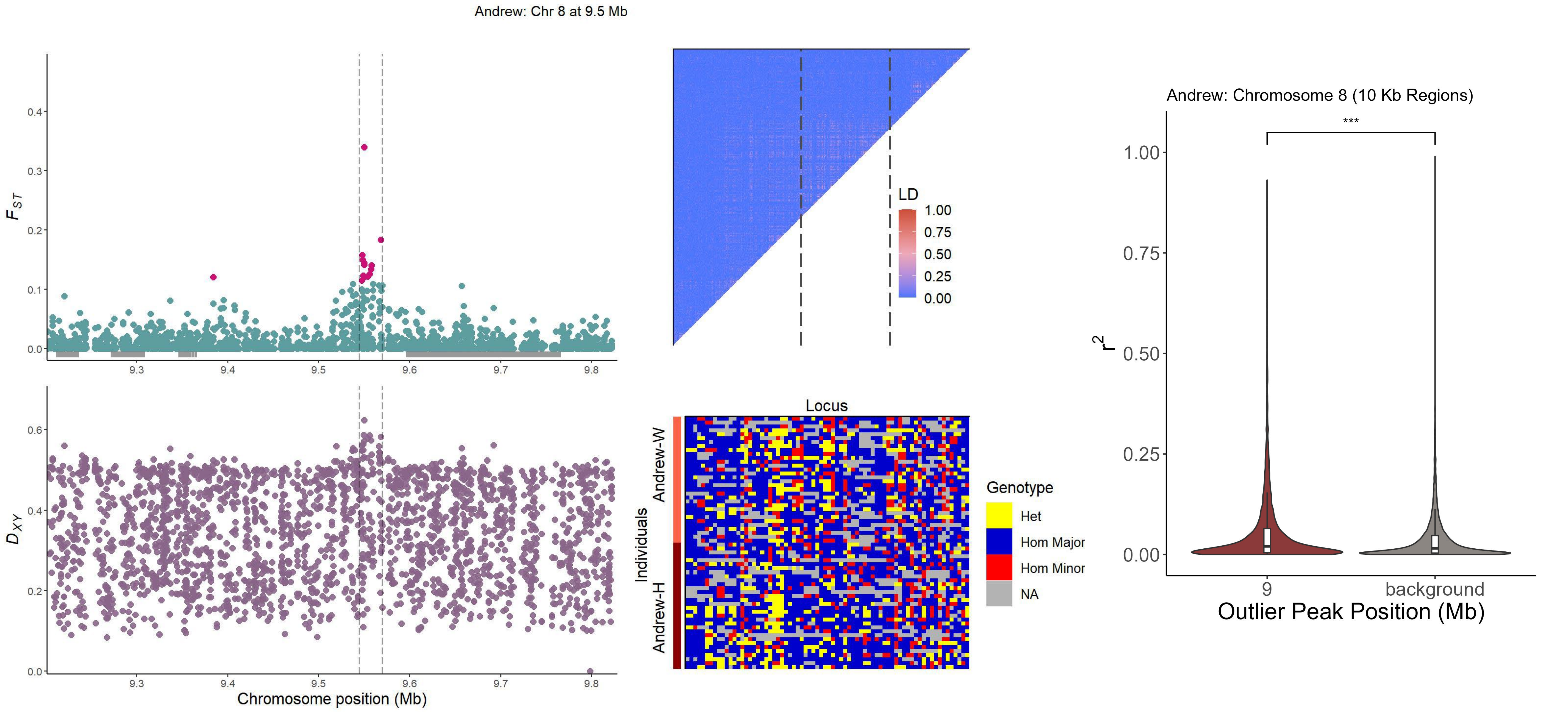
Andrew: Chr 8 at 9.5 Mb
F_{ST}
0.4
0.3
0.2
0.1
0.0
9.3
9.4
9.5
9.6
9.7
9.8
D_{XY}
0.6
0.4
0.2
0.0
Chromosome position (Mb)
LD
1.00
0.75
0.50
0.25
0.00
Locus
Individuals
Andrew-W
Andrew-H
Genotype
Het
Hom Major
Hom Minor
NA
Andrew: Chromosome 8 (10 Kb Regions)
***
r^2
1.00
0.75
0.50
0.25
0.00
9
background
Outlier Peak Position (Mb)

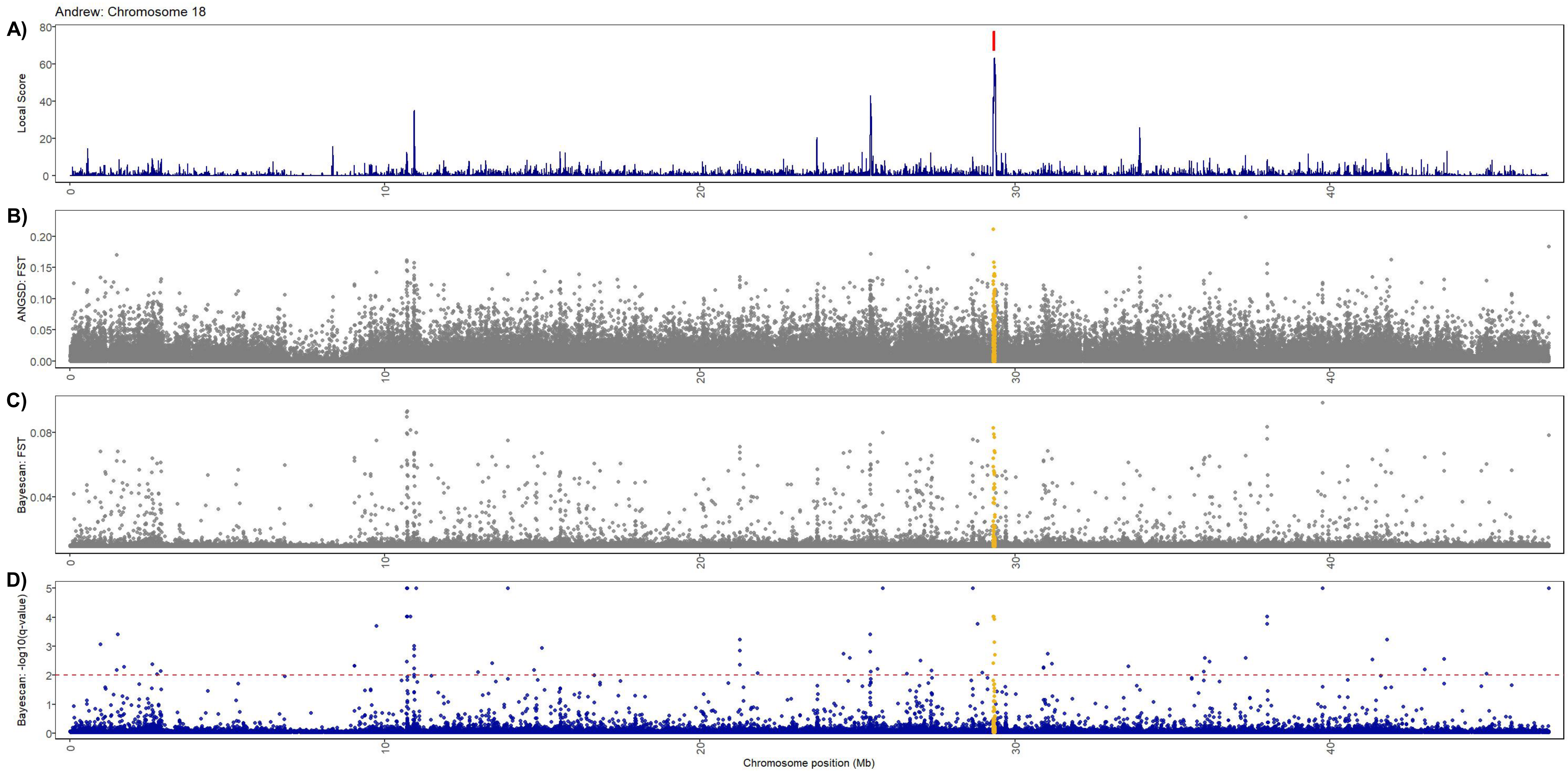

Manhattan plots for Andrew H-W comparison on chromosome 18. A) Local score plot with the red rectangle signifying an outlier peak identified through local score; B) FST calculated in ANGSD; C) FST calculated in Bayescan; D) –log(q-value) calculated in Bayescan with the red dashed line signifying the cutoff for outlier loci expected to be under selection. The yellow points in panels B – D are loci within the local score outlier peak boundaries.

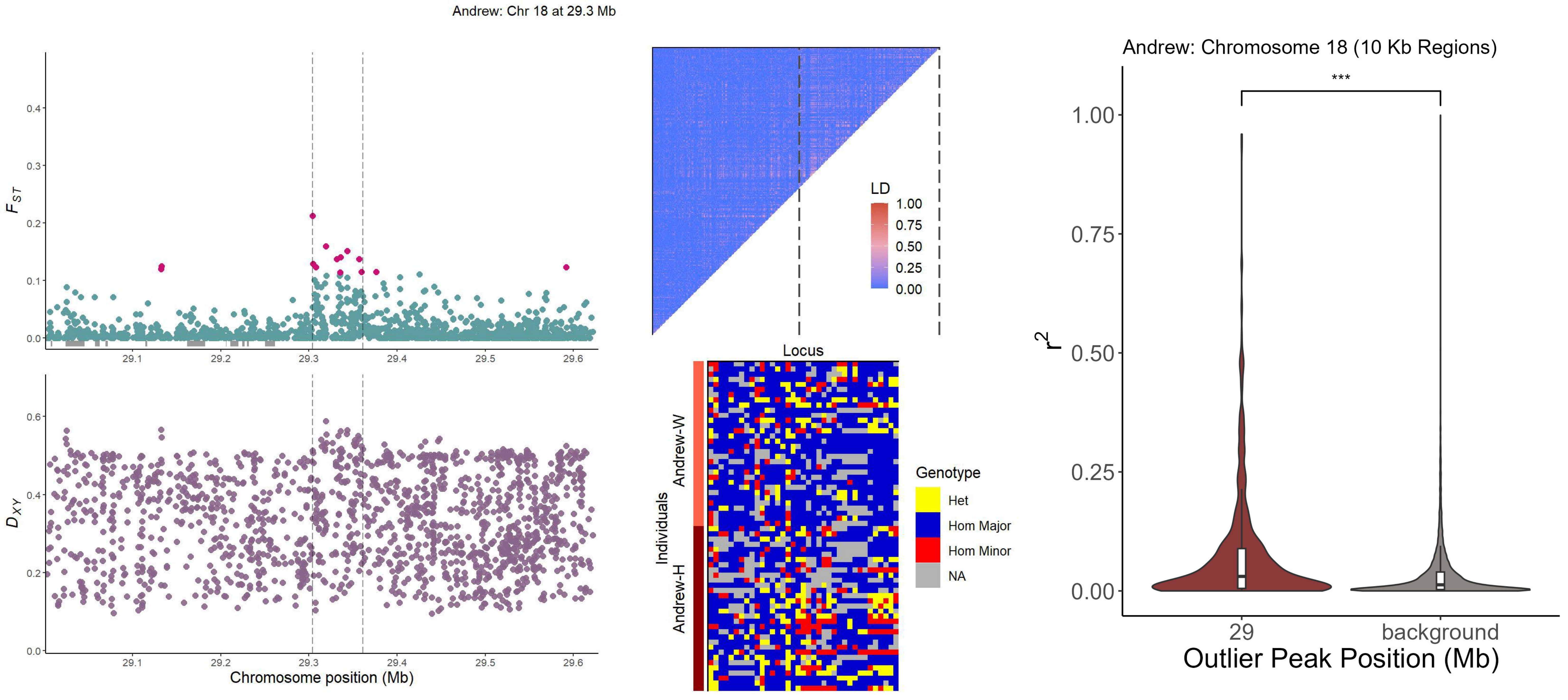

Andrew: Chr 18 at 29.3 Mb
F_{ST}
D_{XY}
Chromosome position (Mb)
LD
1.00
0.75
0.50
0.25
0.00
Locus
Individuals
Andrew-W
Andrew-H
Genotype
Het
Hom Major
Hom Minor
NA
Andrew: Chromosome 18 (10 Kb Regions)
***
r^2
29
background
Outlier Peak Position (Mb)

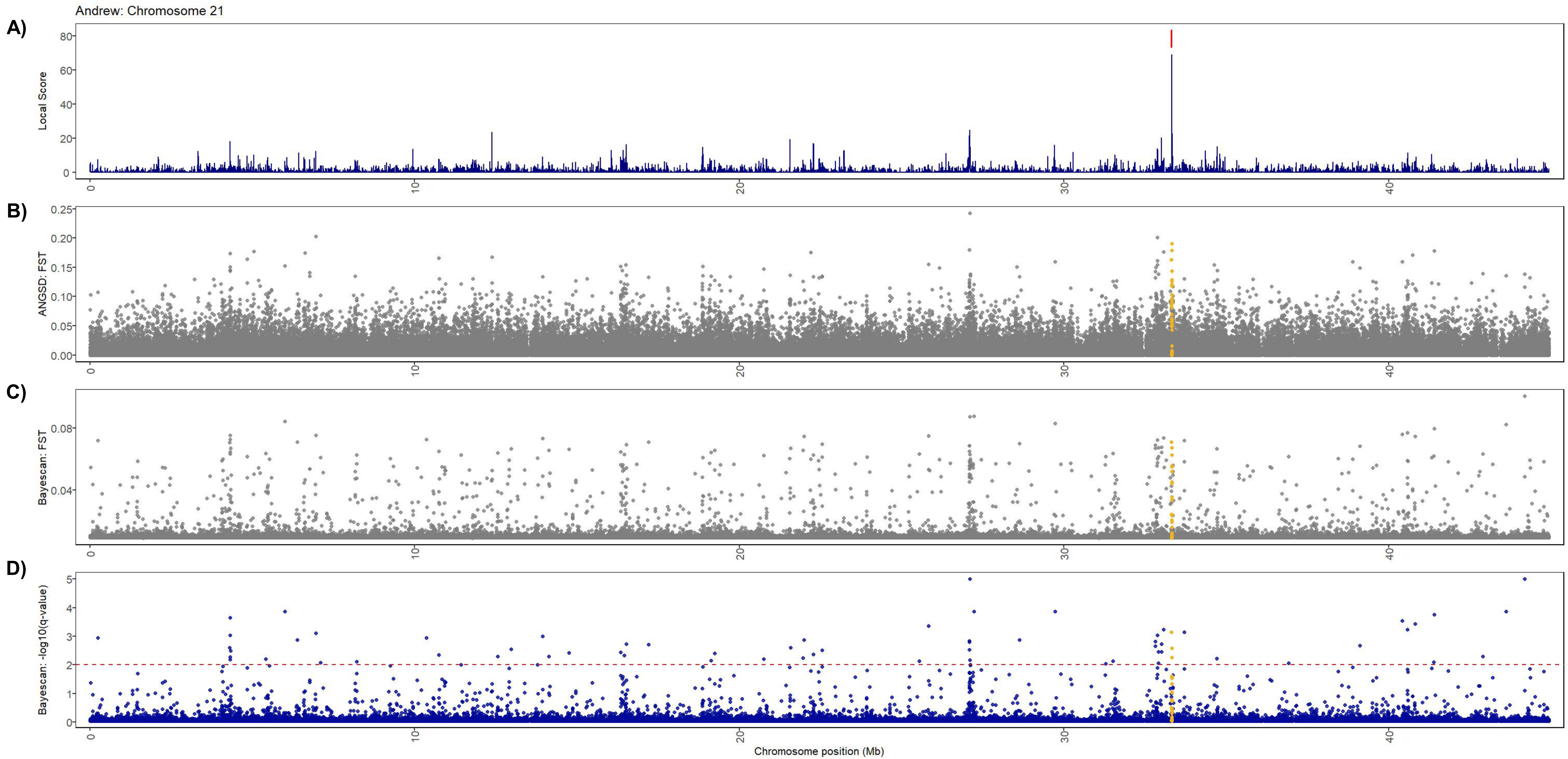

Manhattan plots for Andrew H-W comparison on chromosome 21. A) Local score plot with the red rectangle signifying an outlier peak identified through local score; B) FST calculated in ANGSD; C) FST calculated in Bayescan; D) –log(q-value) calculated in Bayescan with the red dashed line signifying the cutoff for outlier loci expected to be under selection. The yellow points in panels B – D are loci within the local score outlier peak boundaries.

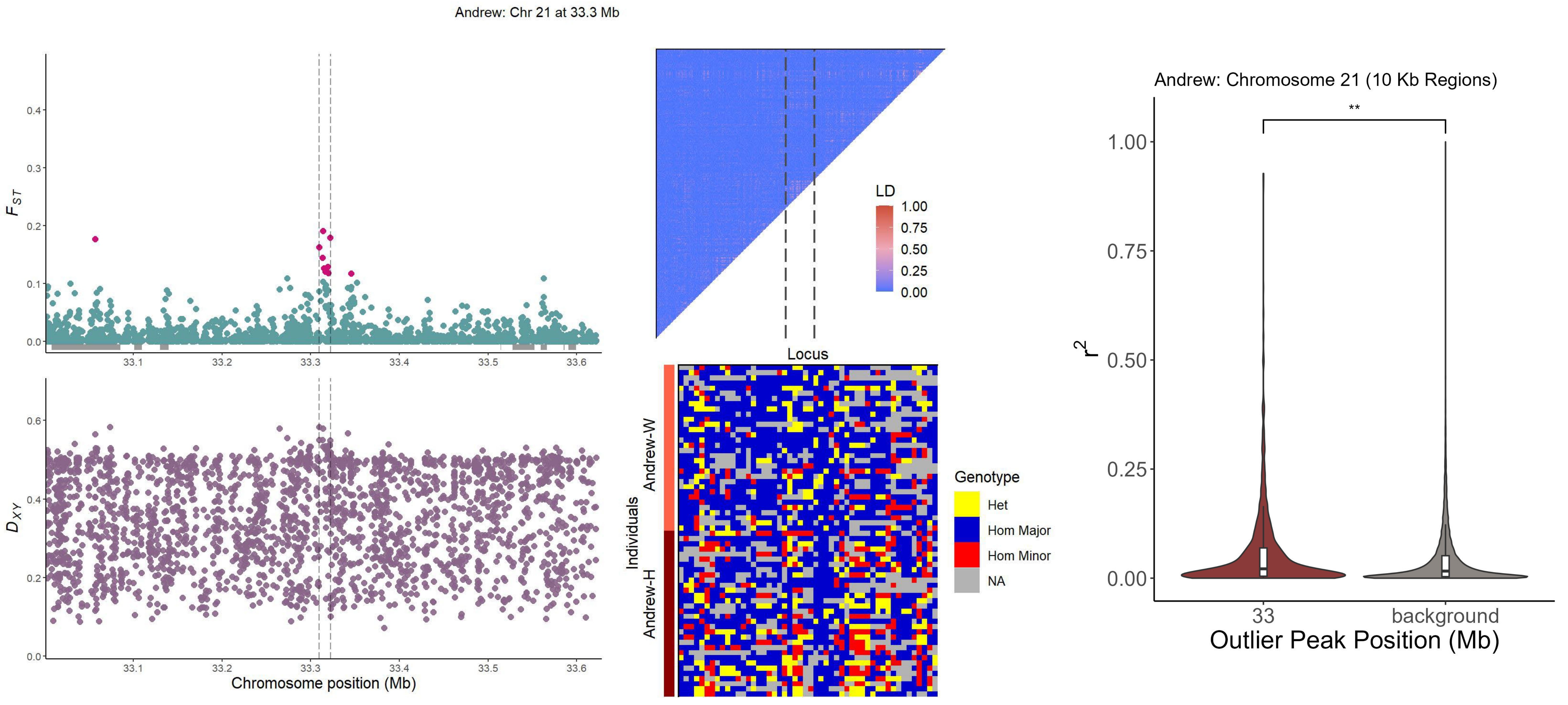

Andrew: Chr 21 at 33.3 Mb
F_{ST}
0.4
0.3
0.2
0.1
0.0
33.1
33.2
33.3
33.4
33.5
33.6
D_{XY}
0.6
0.4
0.2
0.0
Chromosome position (Mb)
LD
1.00
0.75
0.50
0.25
0.00
Locus
Individuals
Andrew-W
Andrew-H
Genotype
Het
Hom Major
Hom Minor
NA
Andrew: Chromosome 21 (10 Kb Regions)
**
r^2
1.00
0.75
0.50
0.25
0.00
33
background
Outlier Peak Position (Mb)

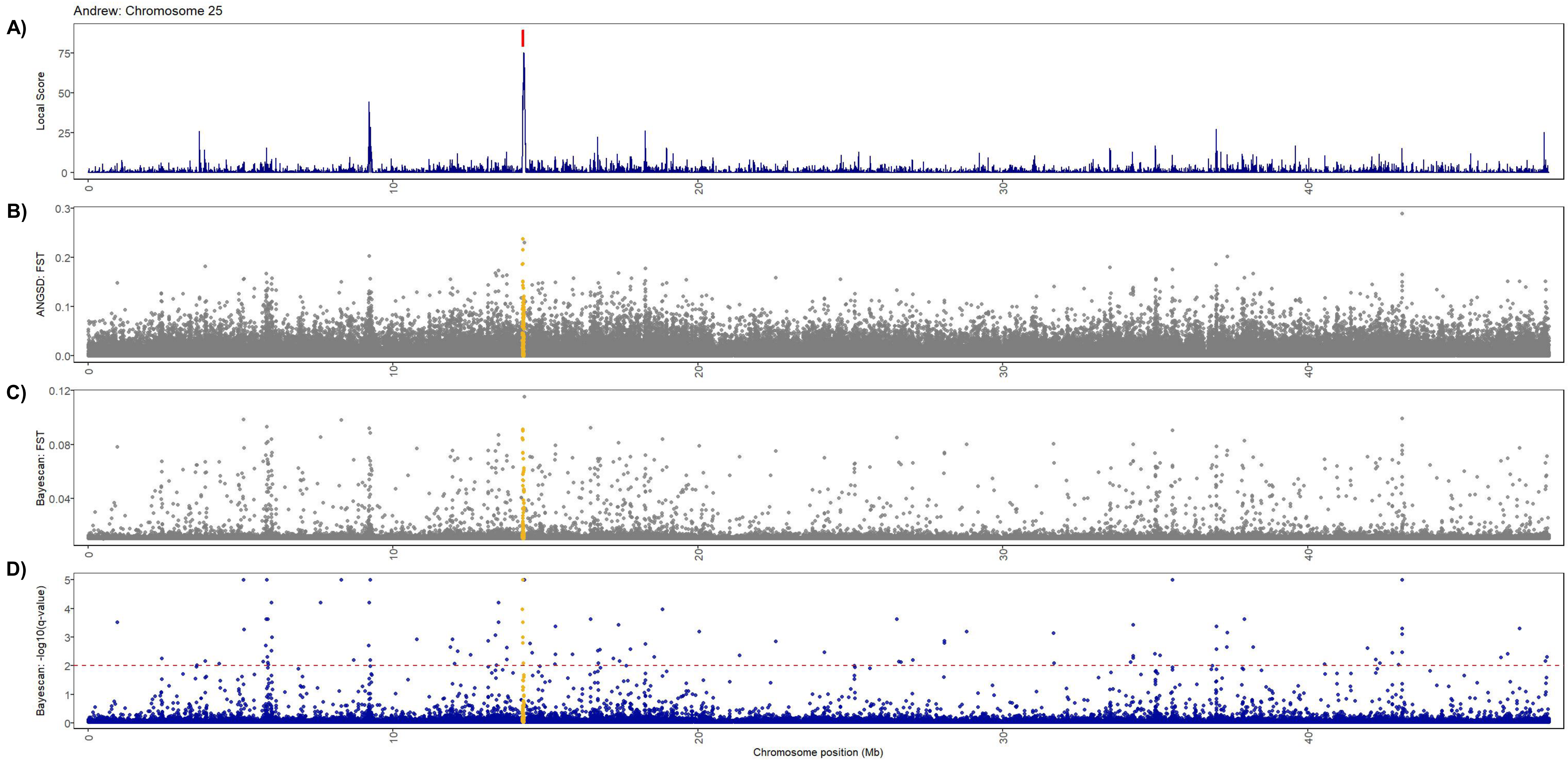

Manhattan plots for Andrew H-W comparison on chromosome 25. A) Local score plot with the red rectangle signifying an outlier peak identified through local score; B) FST calculated in ANGSD; C) FST calculated in Bayescan; D) –log(q-value) calculated in Bayescan with the red dashed line signifying the cutoff for outlier loci expected to be under selection. The yellow points in panels B – D are loci within the local score outlier peak boundaries.

Andrew: Chr 25 at 14.2 Mb

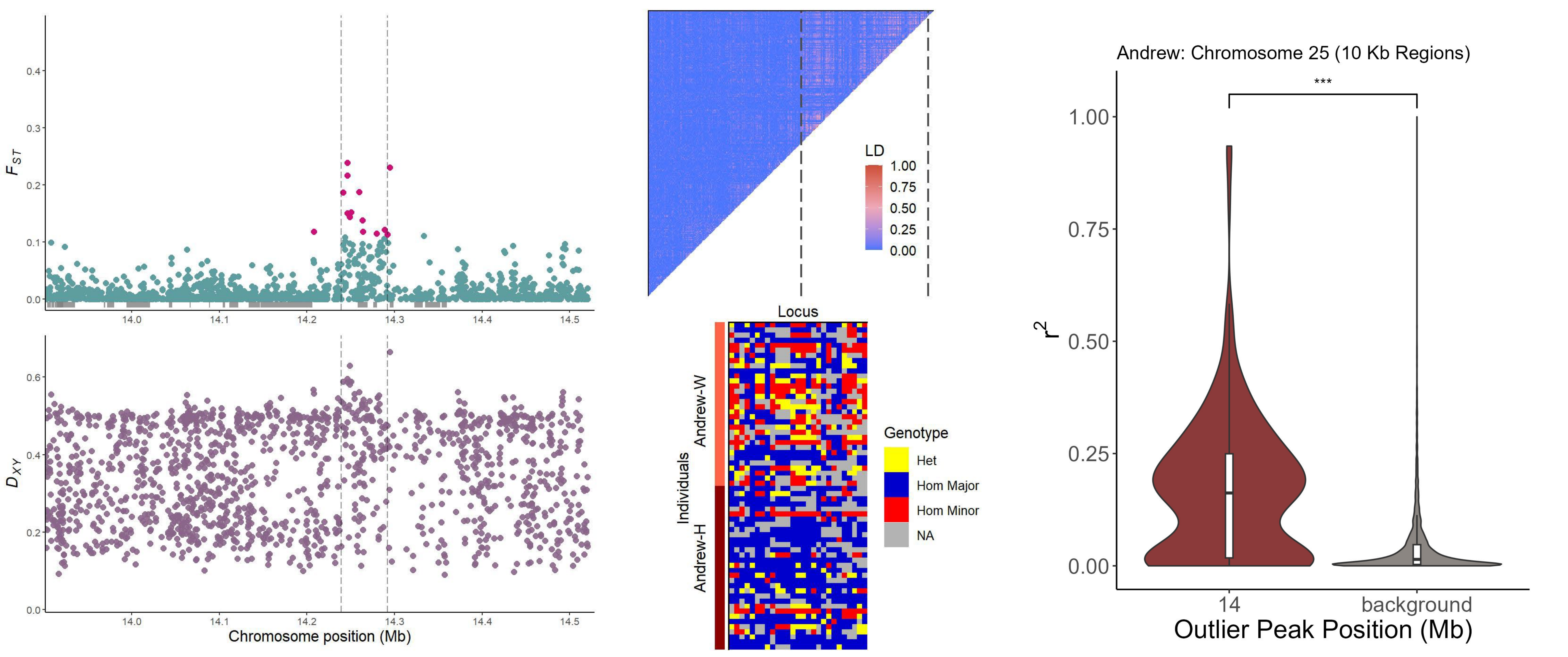

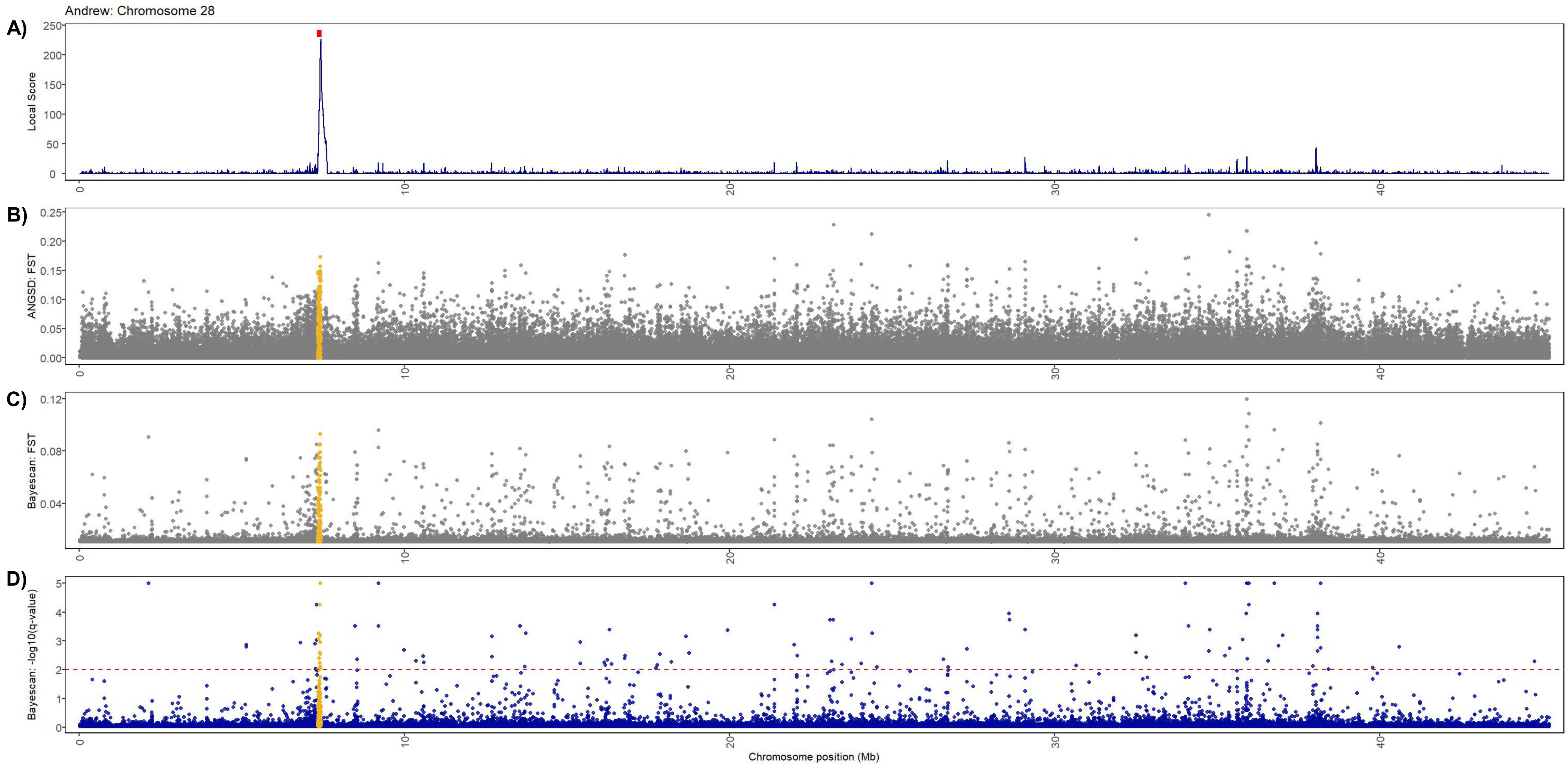

Manhattan plots for Andrew H-W comparison on chromosome 28. A) Local score plot with the red rectangle signifying an outlier peak identified through local score; B) FST calculated in ANGSD; C) FST calculated in Bayescan; D) –log(q-value) calculated in Bayescan with the red dashed line signifying the cutoff for outlier loci expected to be under selection. The yellow points in panels B – D are loci within the local score outlier peak boundaries.

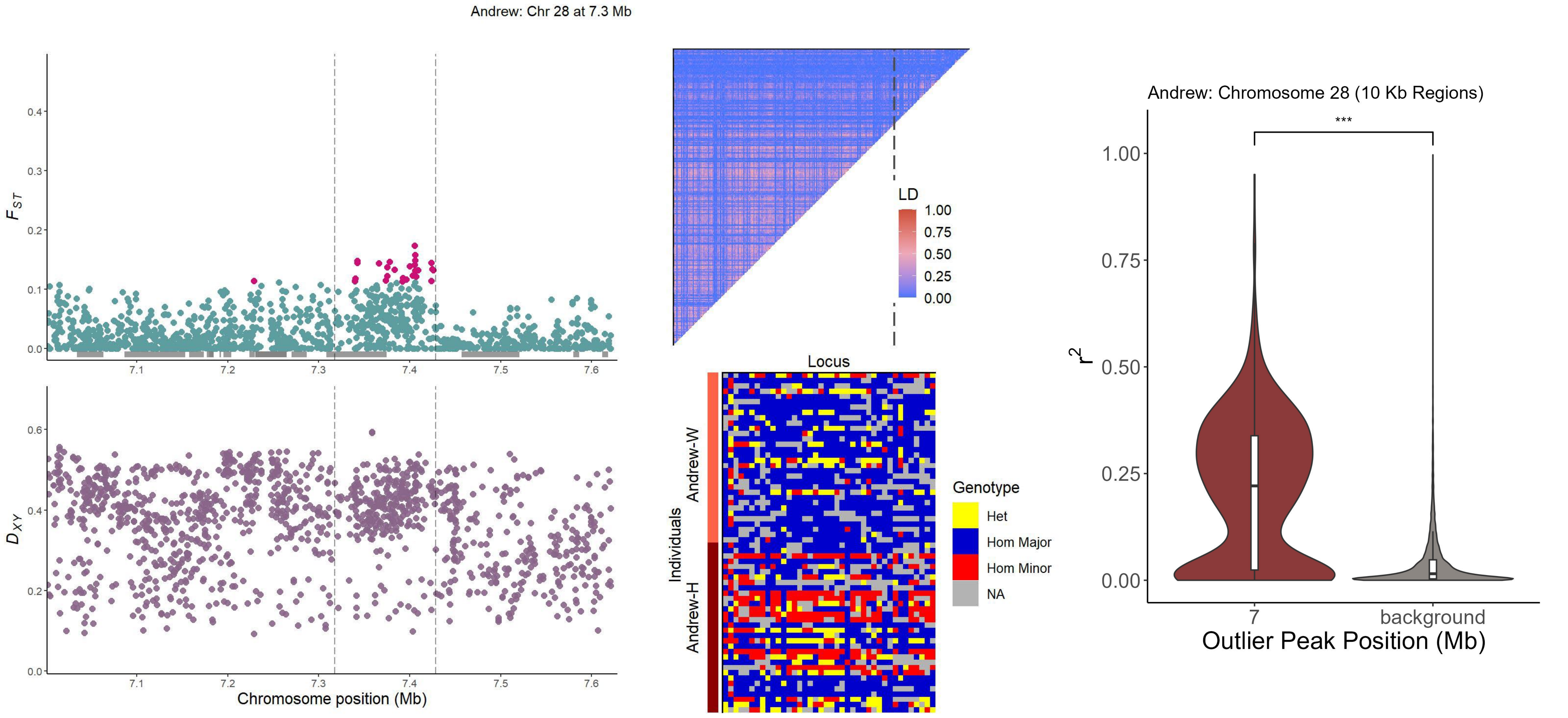

Andrew: Chr 28 at 7.3 Mb
F_{ST}
D_{XY}
Chromosome position (Mb)
LD
Locus
Individuals
Andrew-W
Andrew-H
Genotype
Het
Hom Major
Hom Minor
NA
Andrew: Chromosome 28 (10 Kb Regions)
***
r^2
7
background
Outlier Peak Position (Mb)

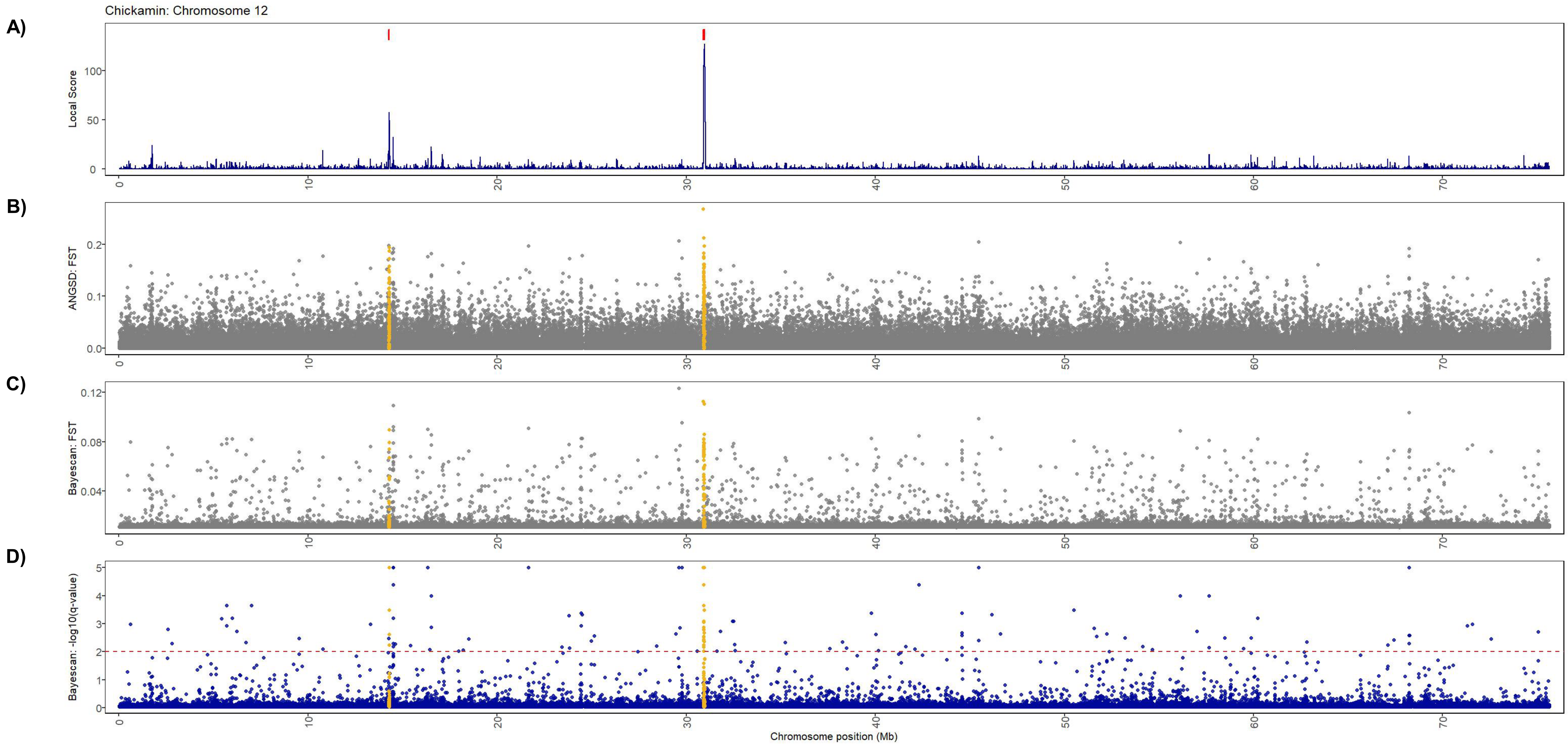

Manhattan plots for Chickamin H-W comparison on chromosome 12. A) Local score plot with the red rectangle signifying an outlier peak identified through local score; B) FST calculated in ANGSD; C) FST calculated in Bayescan; D) –log(q-value) calculated in Bayescan with the red dashed line signifying the cutoff for outlier loci expected to be under selection. The yellow points in panels B – D are loci within the local score outlier peak boundaries.

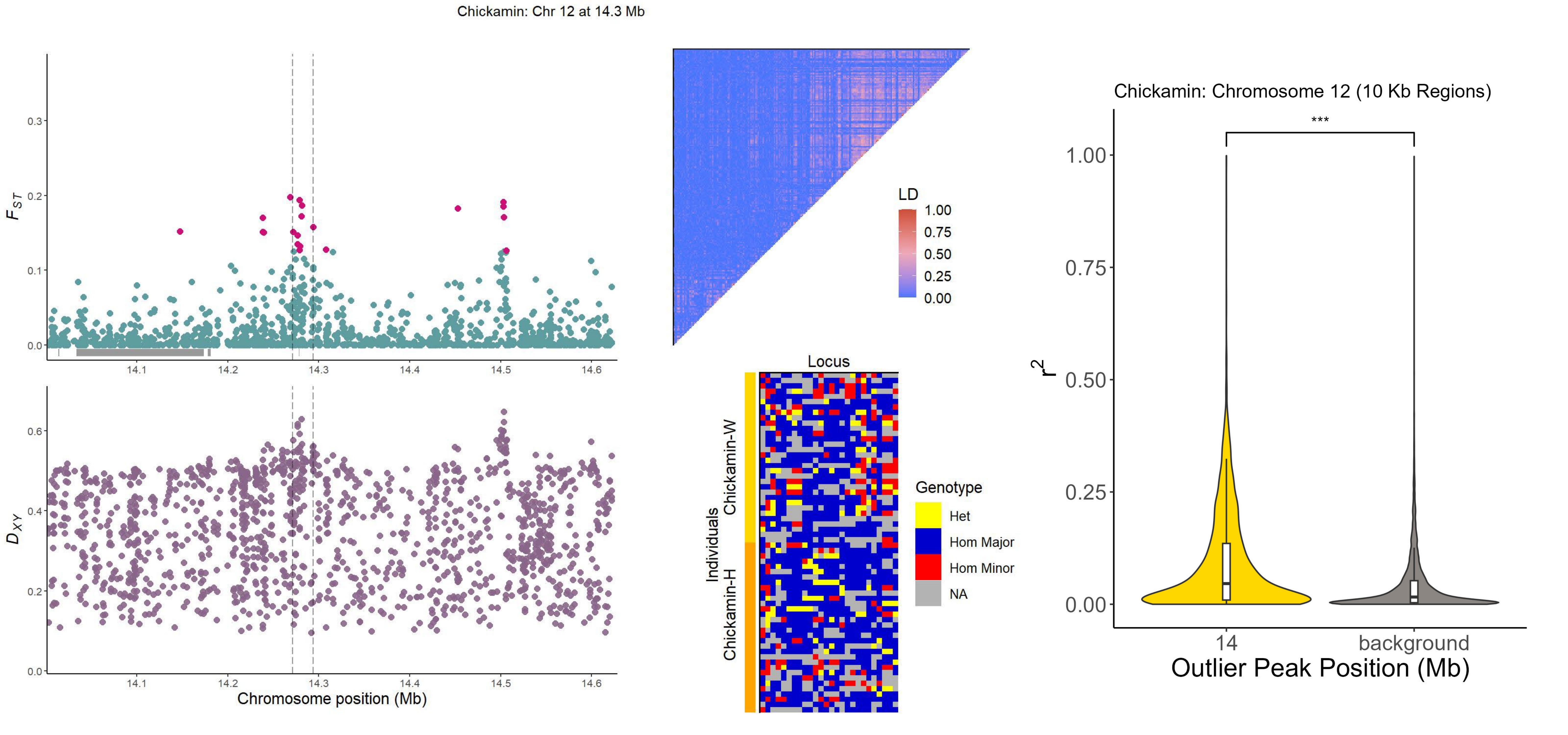

Chickamin: Chr 12 at 14.3 Mb
F_ST
D_XY
Chromosome position (Mb)
0.0
0.1
0.2
0.3
0.4
0.6
14.1
14.2
14.3
14.4
14.5
14.6
LD
1.00
0.75
0.50
0.25
0.00
Locus
Individuals
Chickamin-W
Chickamin-H
Genotype
Het
Hom Major
Hom Minor
NA
Chickamin: Chromosome 12 (10 Kb Regions)
***
r^2
14
background
Outlier Peak Position (Mb)

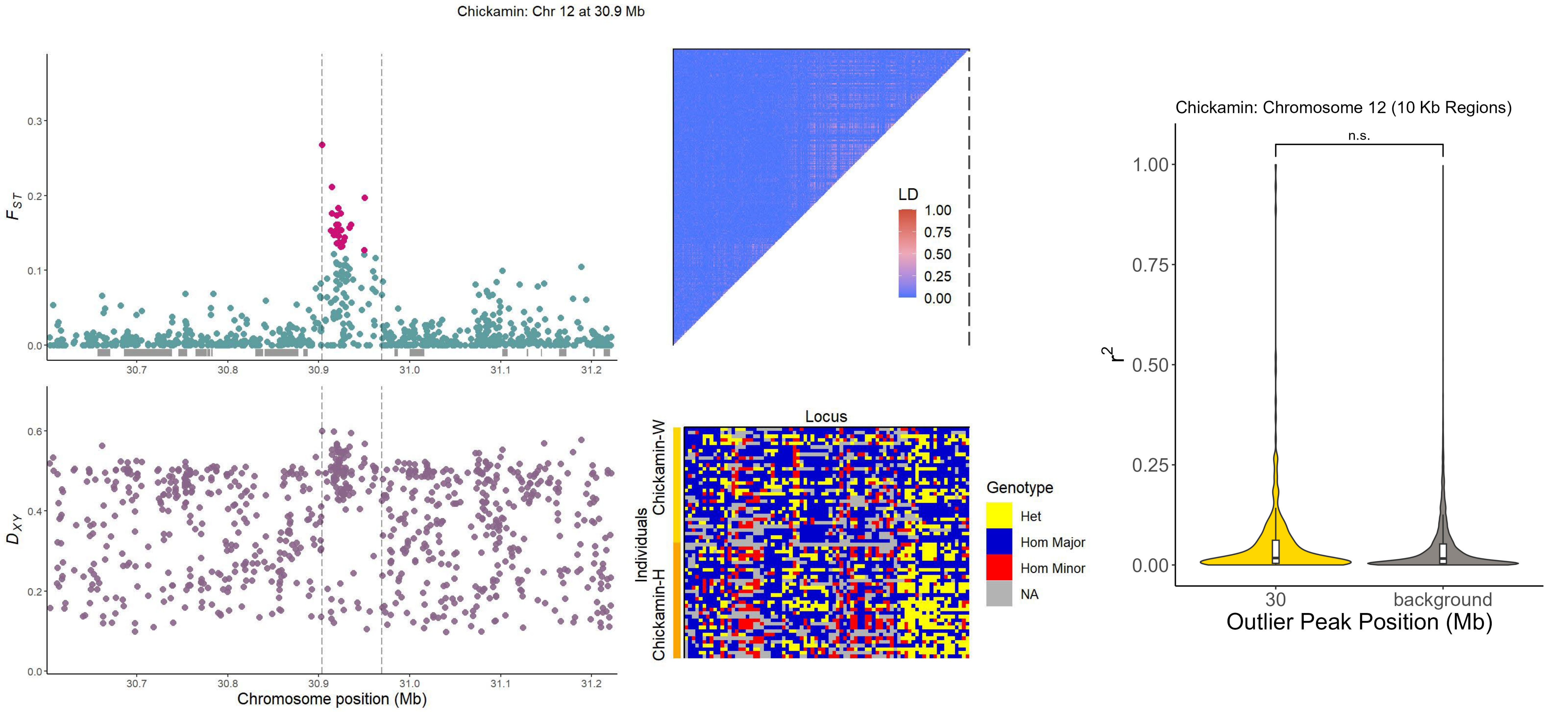
Chickamin: Chr 12 at 30.9 Mb
F_{ST}
D_{XY}
Chromosome position (Mb)
LD
1.00
0.75
0.50
0.25
0.00
Locus
Individuals
Chickamin-W
Chickamin-H
Genotype
Het
Hom Major
Hom Minor
NA
Chickamin: Chromosome 12 (10 Kb Regions)
n.s.
r^2
30
background
Outlier Peak Position (Mb)

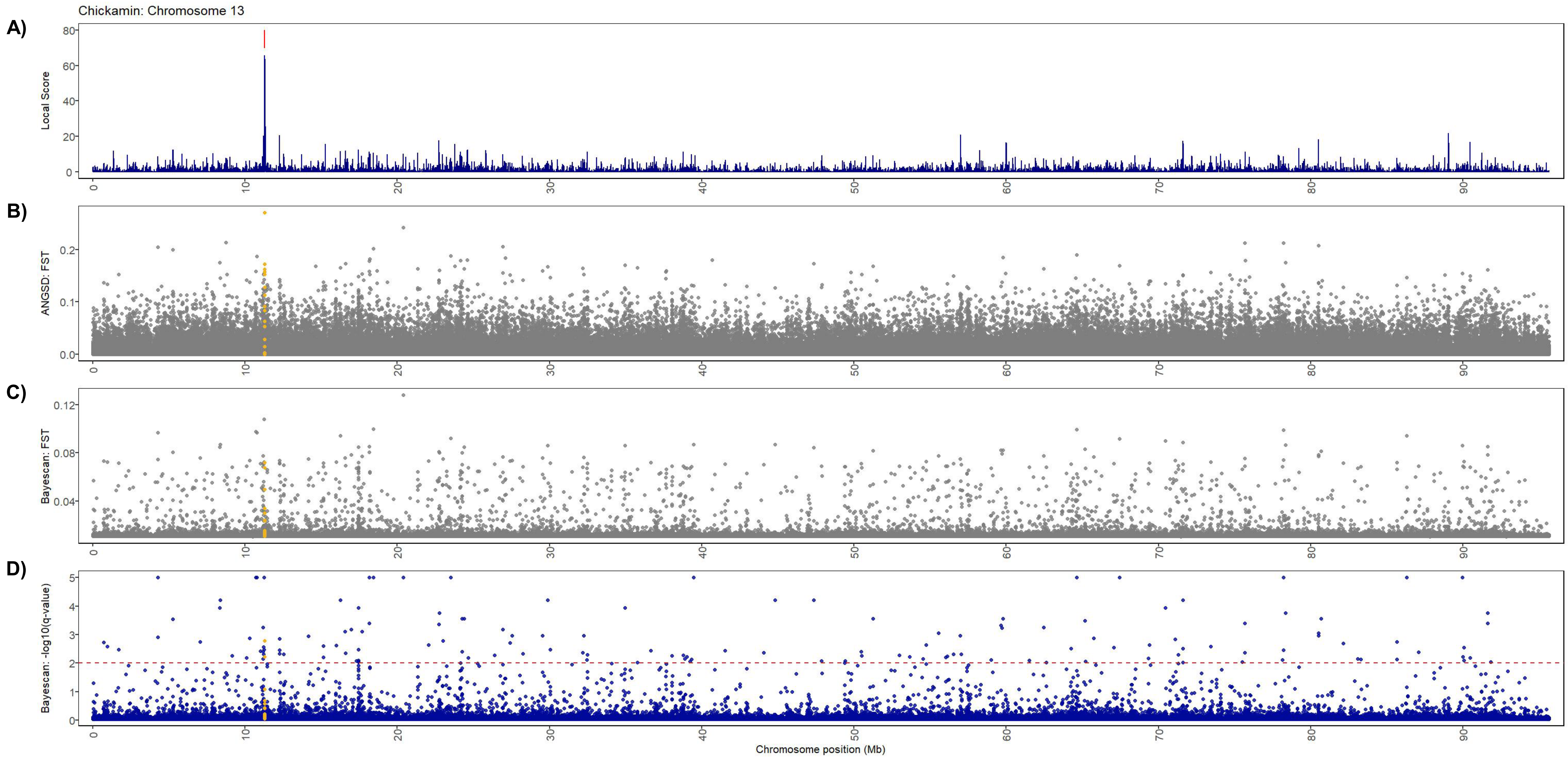

Manhattan plots for Chickamin H-W comparison on chromosome 13. A) Local score plot with the red rectangle signifying an outlier peak identified through local score; B) FST calculated in ANGSD; C) FST calculated in Bayescan; D) –log(q-value) calculated in Bayescan with the red dashed line signifying the cutoff for outlier loci expected to be under selection. The yellow points in panels B – D are loci within the local score outlier peak boundaries.

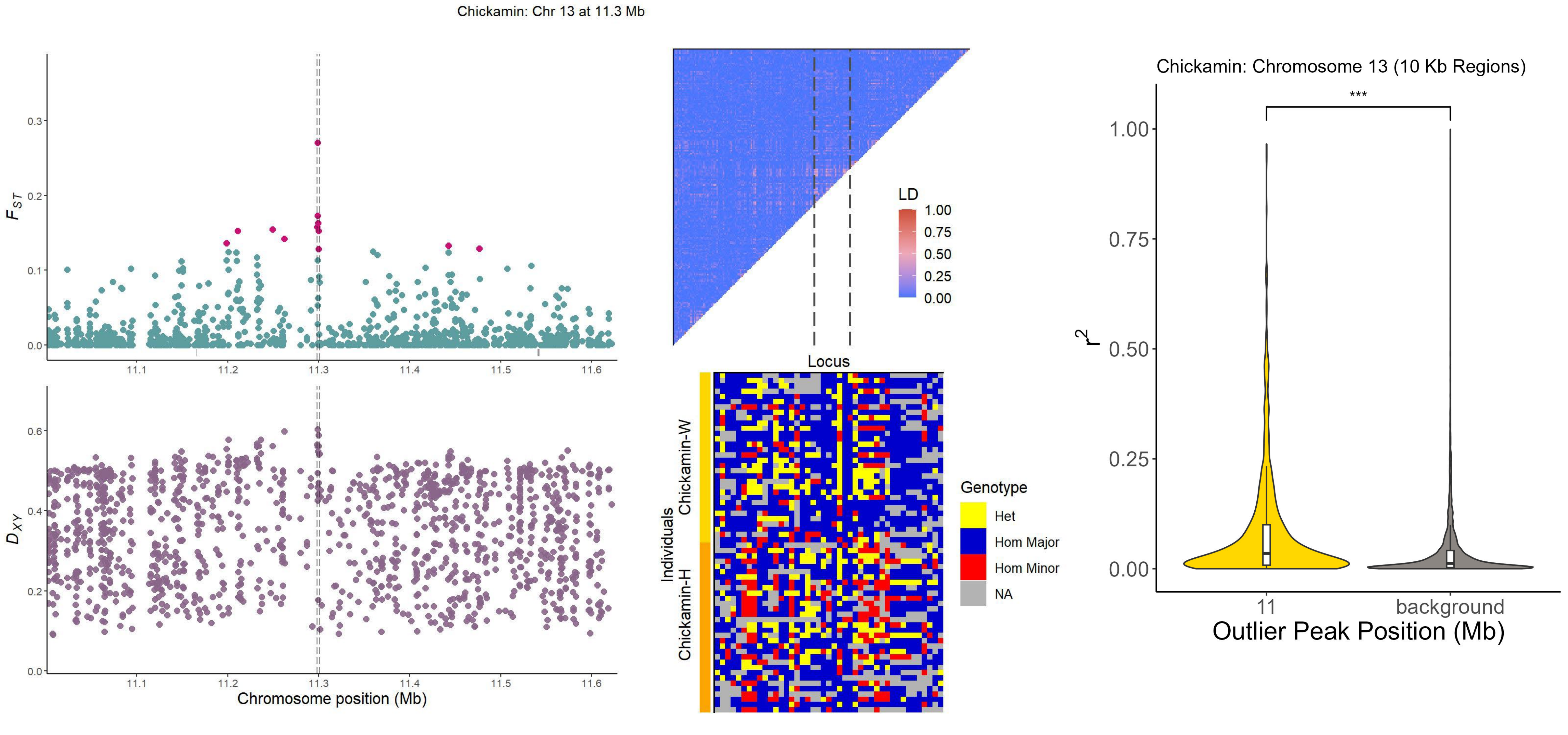

Chickamin: Chr 13 at 11.3 Mb
FST
DXY
Chromosome position (Mb)
0.0
0.1
0.2
0.3
0.4
0.6
11.1
11.2
11.3
11.4
11.5
11.6
LD
1.00
0.75
0.50
0.25
0.00
Locus
Individuals
Chickamin-W
Chickamin-H
Genotype
Het
Hom Major
Hom Minor
NA
Chickamin: Chromosome 13 (10 Kb Regions)
***
r2
11
background
Outlier Peak Position (Mb)

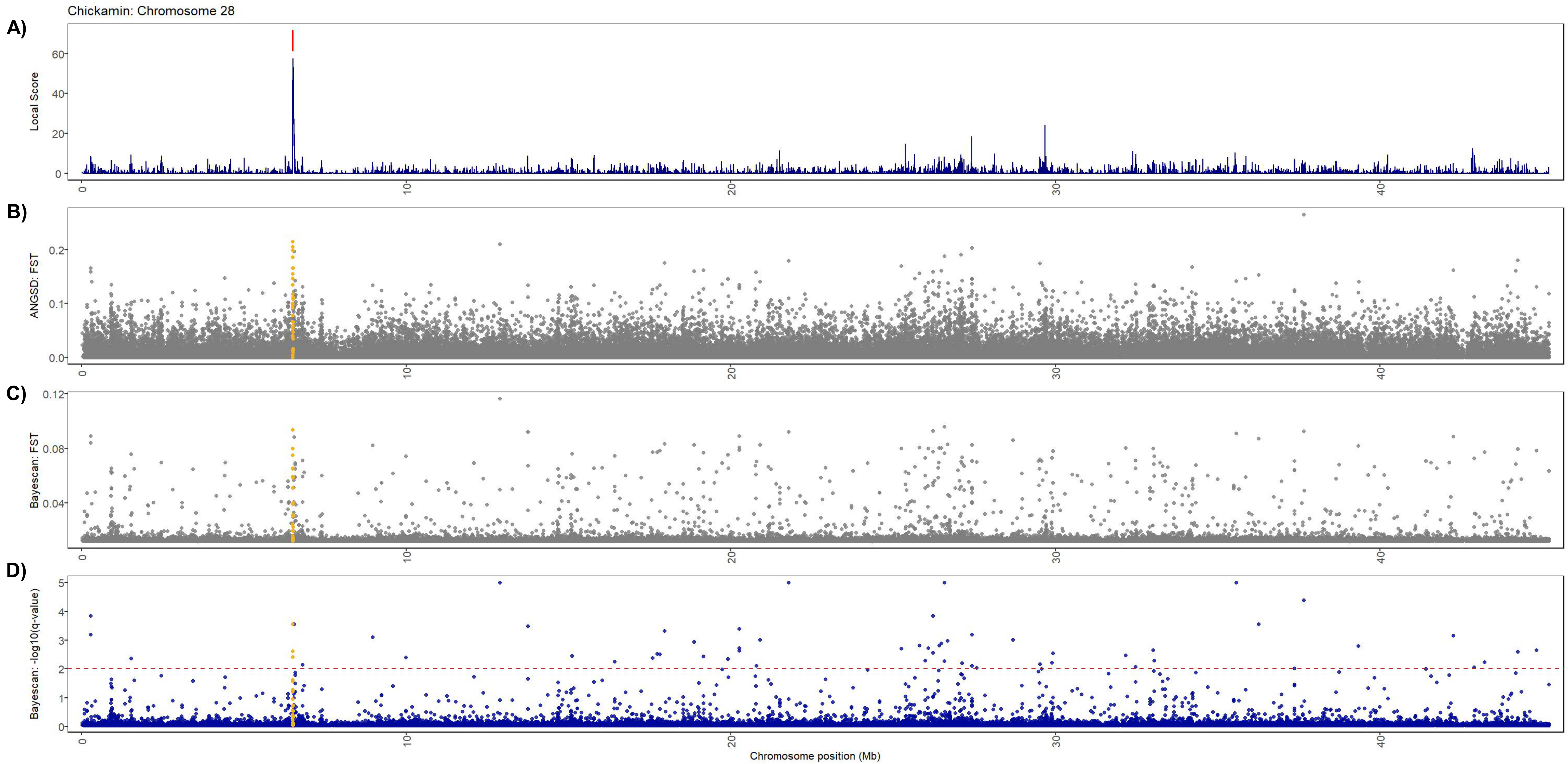

Manhattan plots for Chickamin H-W comparison on chromosome 28. A) Local score plot with the red rectangle signifying an outlier peak identified through local score; B) FST calculated in ANGSD; C) FST calculated in Bayescan; D) –log(q-value) calculated in Bayescan with the red dashed line signifying the cutoff for outlier loci expected to be under selection. The yellow points in panels B – D are loci within the local score outlier peak boundaries.

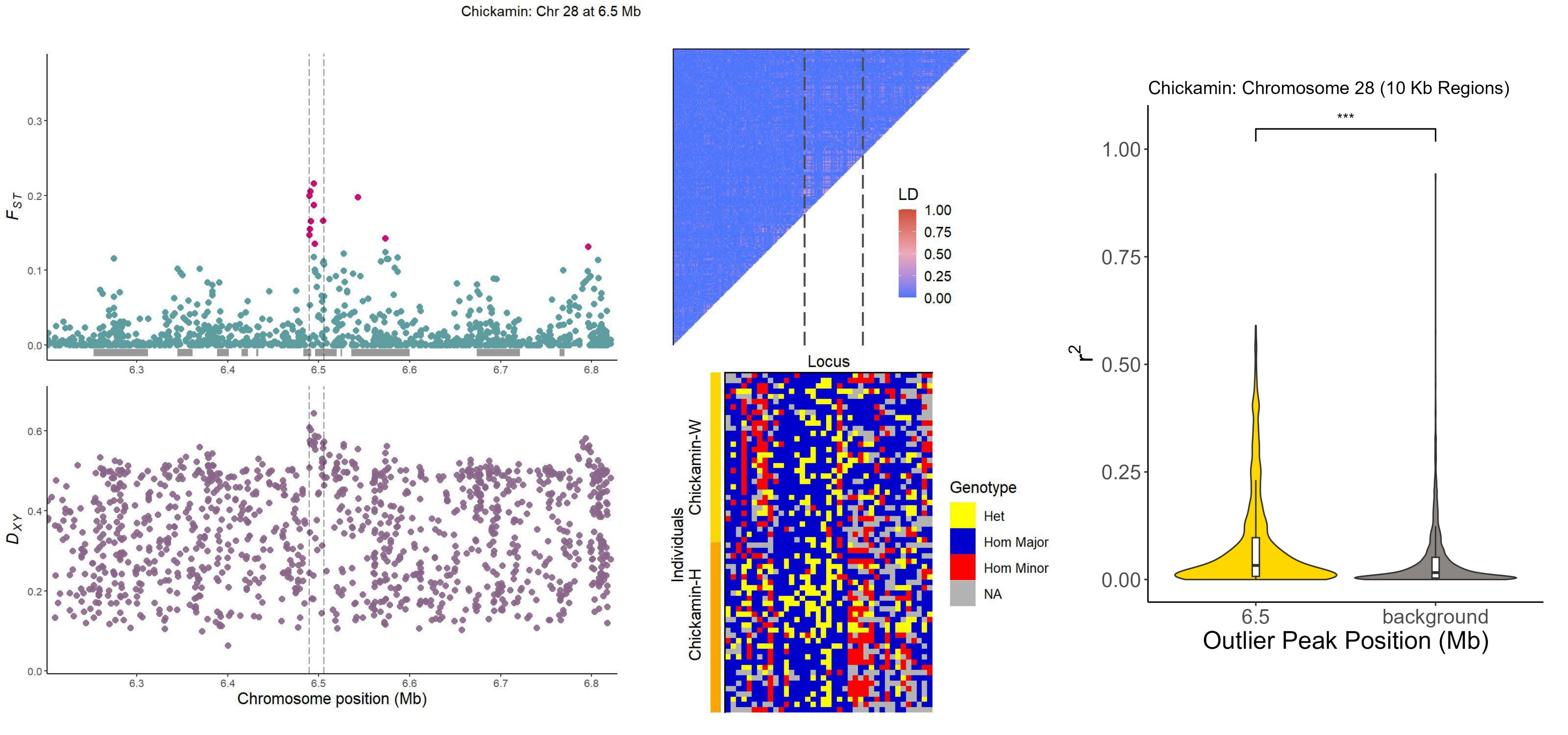

Chickamin: Chr 28 at 6.5 Mb
F_ST
D_XY
Chromosome position (Mb)
LD
1.00
0.75
0.50
0.25
0.00
Locus
Individuals
Chickamin-W
Chickamin-H
Genotype
Het
Hom Major
Hom Minor
NA
Chickamin: Chromosome 28 (10 Kb Regions)
***
r²
6.5
background
Outlier Peak Position (Mb)

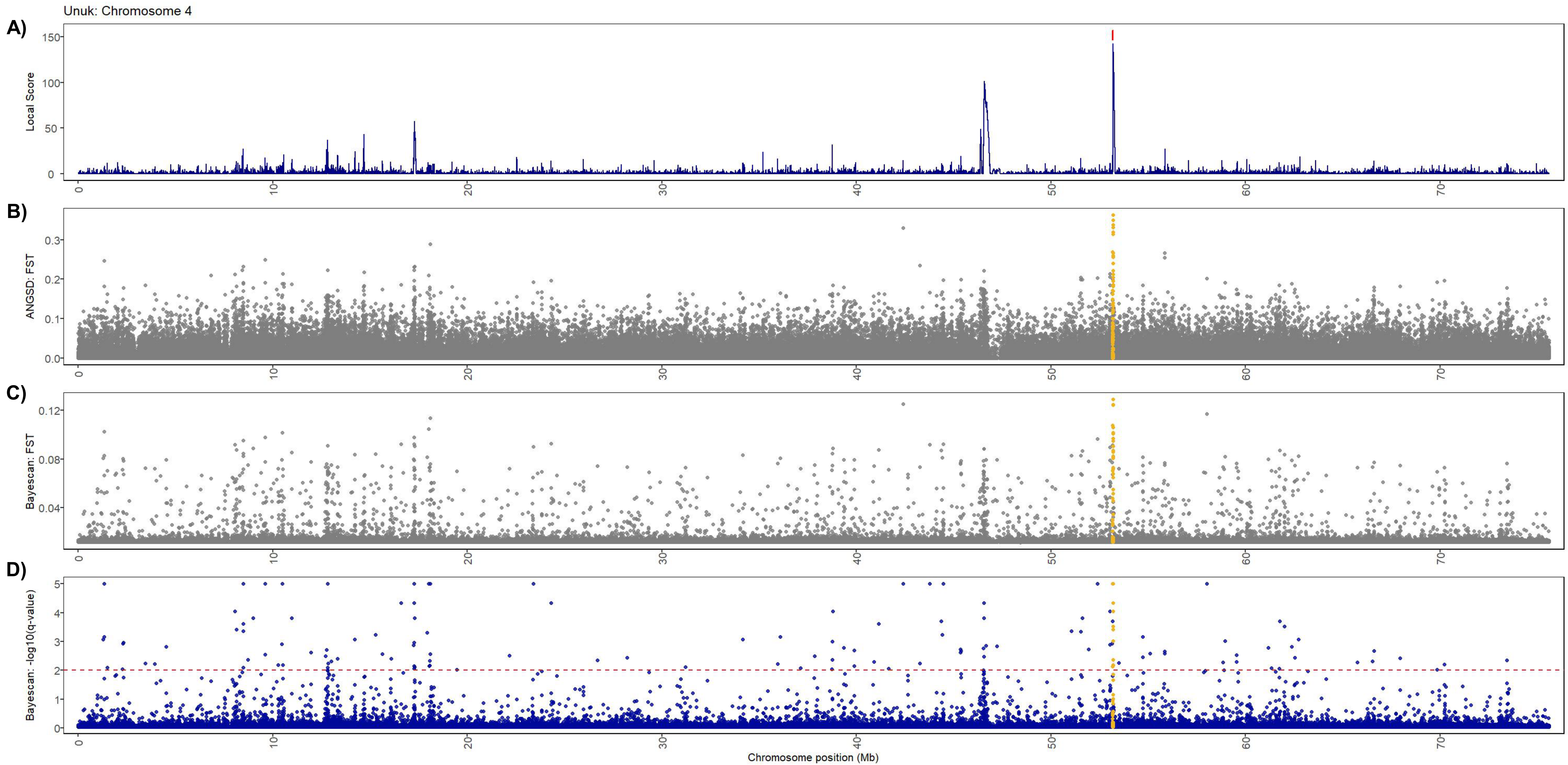

Manhattan plots for Unuk H-W comparison on chromosome 4. A) Local score plot with the red rectangle signifying an outlier peak identified through local score; B) FST calculated in ANGSD; C) FST calculated in Bayescan; D) –log(q-value) calculated in Bayescan with the red dashed line signifying the cutoff for outlier loci expected to be under selection. The yellow points in panels B – D are loci within the local score outlier peak boundaries.

* Both figures in main document (Figures 4 and 5a)

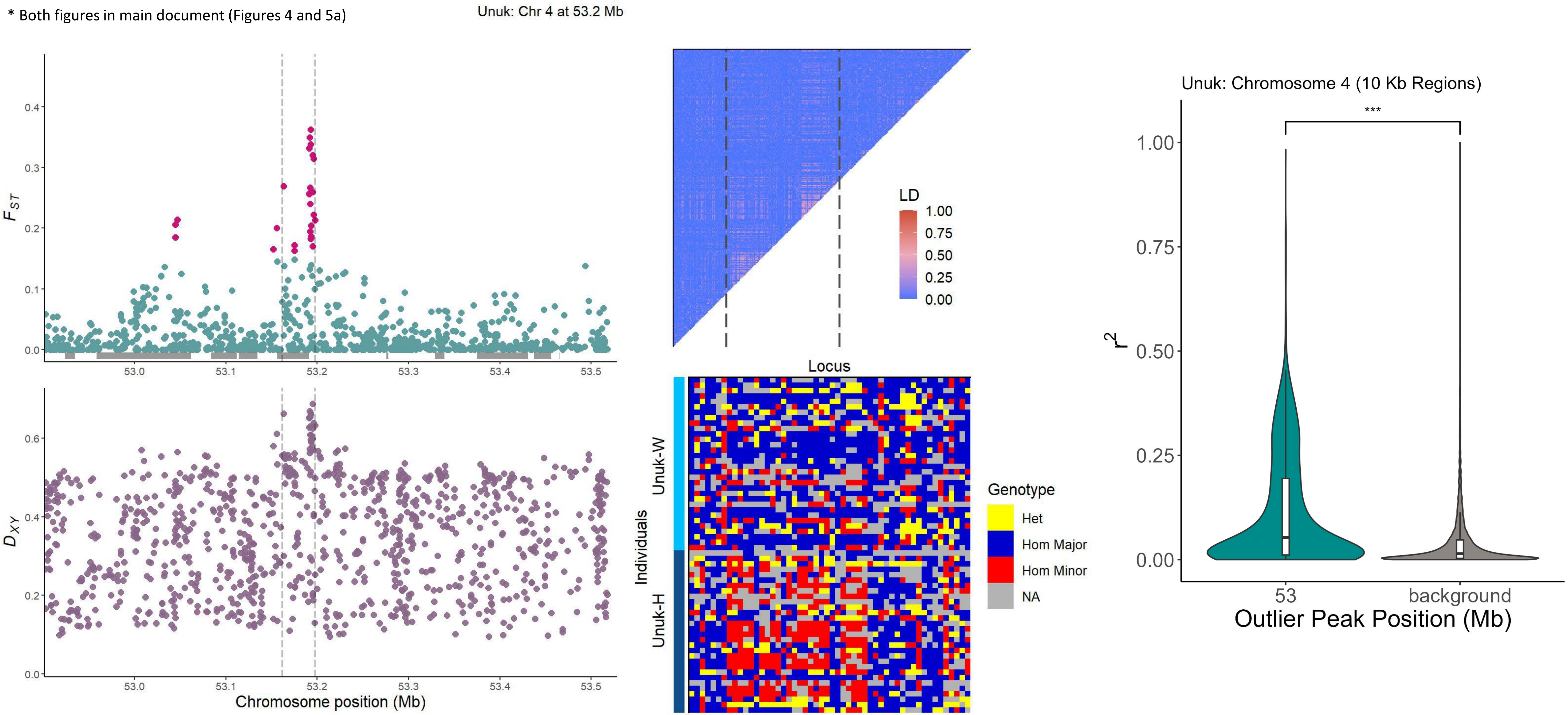

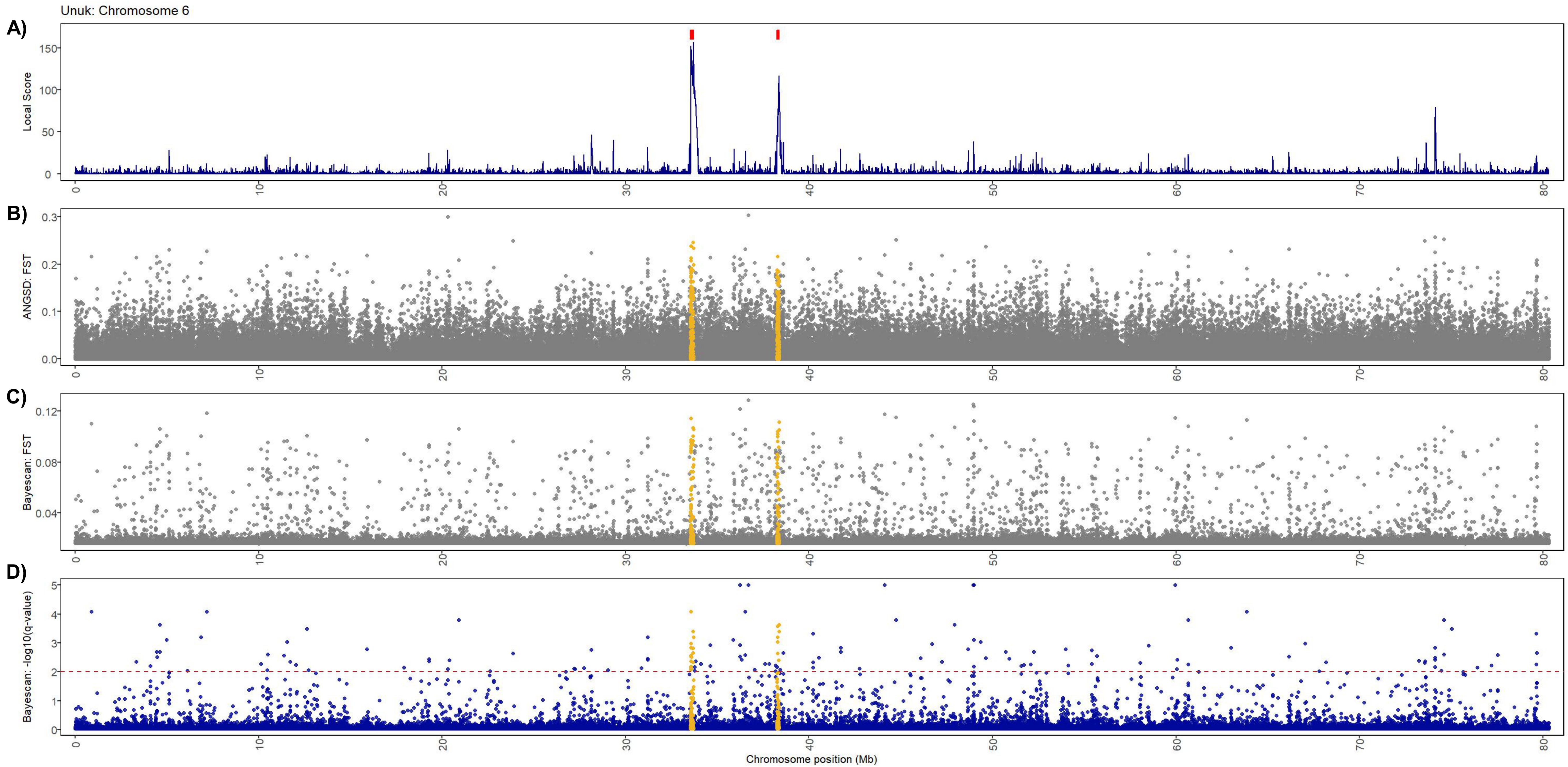

Manhattan plots for Unuk H-W comparison on chromosome 6. A) Local score plot with the red rectangle signifying an outlier peak identified through local score; B) FST calculated in ANGSD; C) FST calculated in Bayescan; D) –log(q-value) calculated in Bayescan with the red dashed line signifying the cutoff for outlier loci expected to be under selection. The yellow points in panels B – D are loci within the local score outlier peak boundaries.

Unuk: Chr 6 at 33.5 Mb

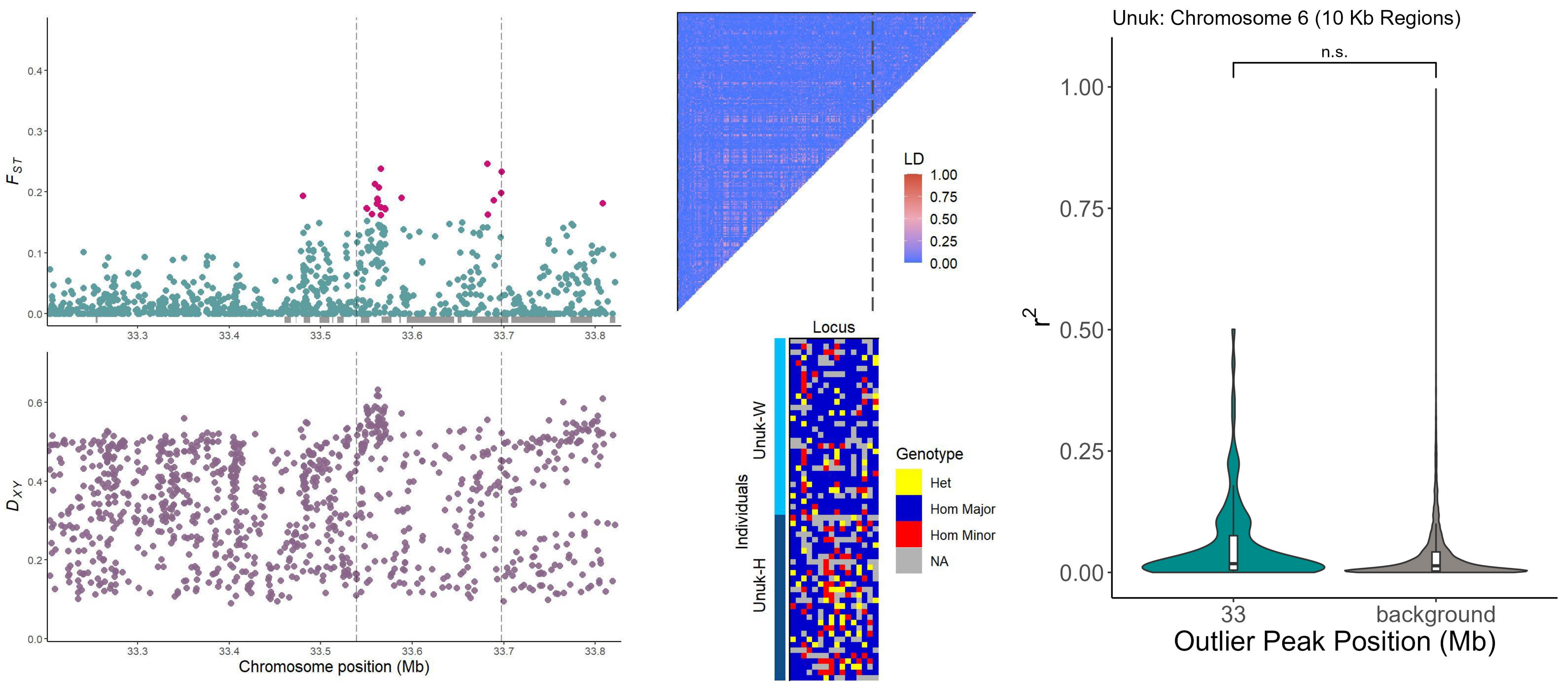

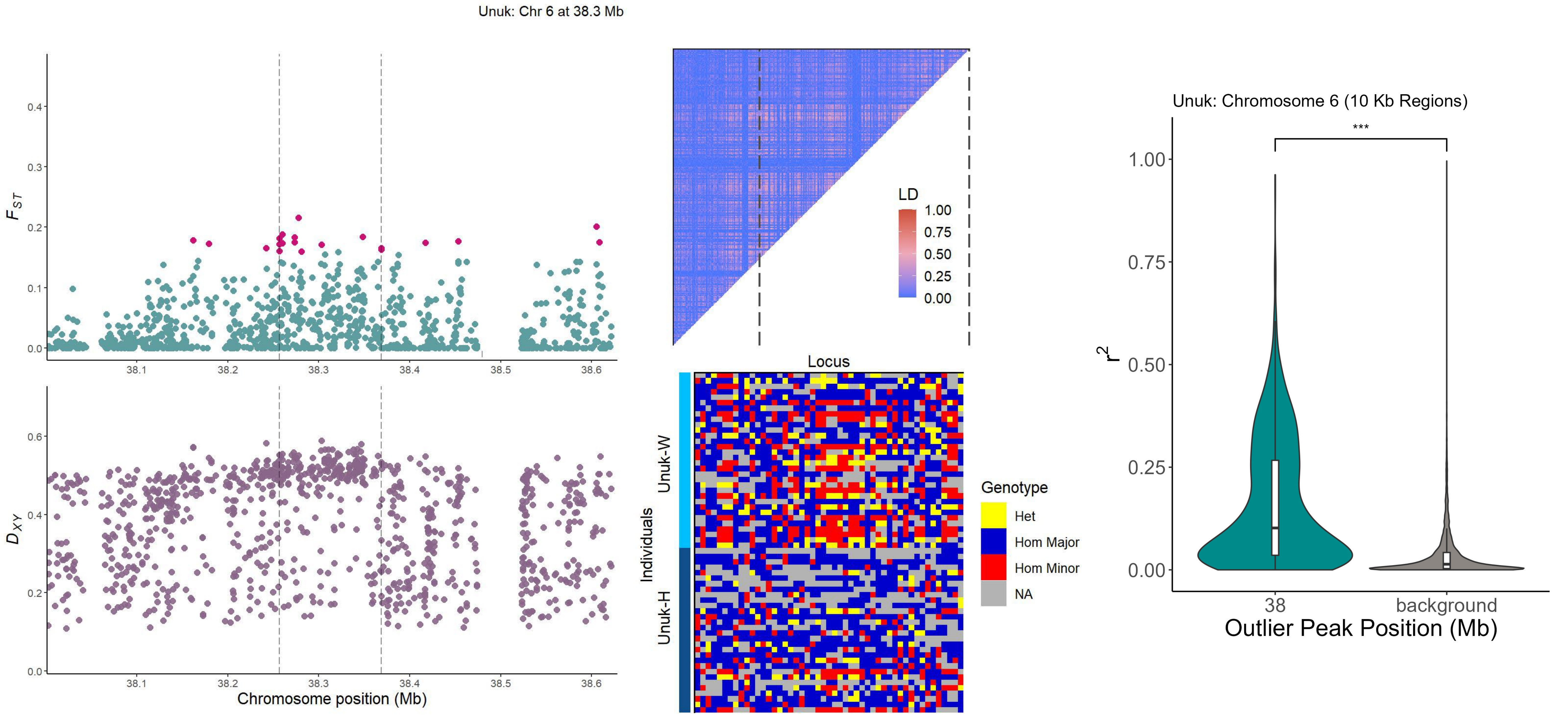

Unuk: Chr 6 at 38.3 Mb
F_{ST}
0.4
0.3
0.2
0.1
0.0
38.1
38.2
38.3
38.4
38.5
38.6
D_{XY}
0.6
0.4
0.2
0.0
Chromosome position (Mb)
LD
1.00
0.75
0.50
0.25
0.00
Locus
Individuals
Unuk-W
Unuk-H
Genotype
Het
Hom Major
Hom Minor
NA
Unuk: Chromosome 6 (10 Kb Regions)
***
r^2
1.00
0.75
0.50
0.25
0.00
38
background
Outlier Peak Position (Mb)

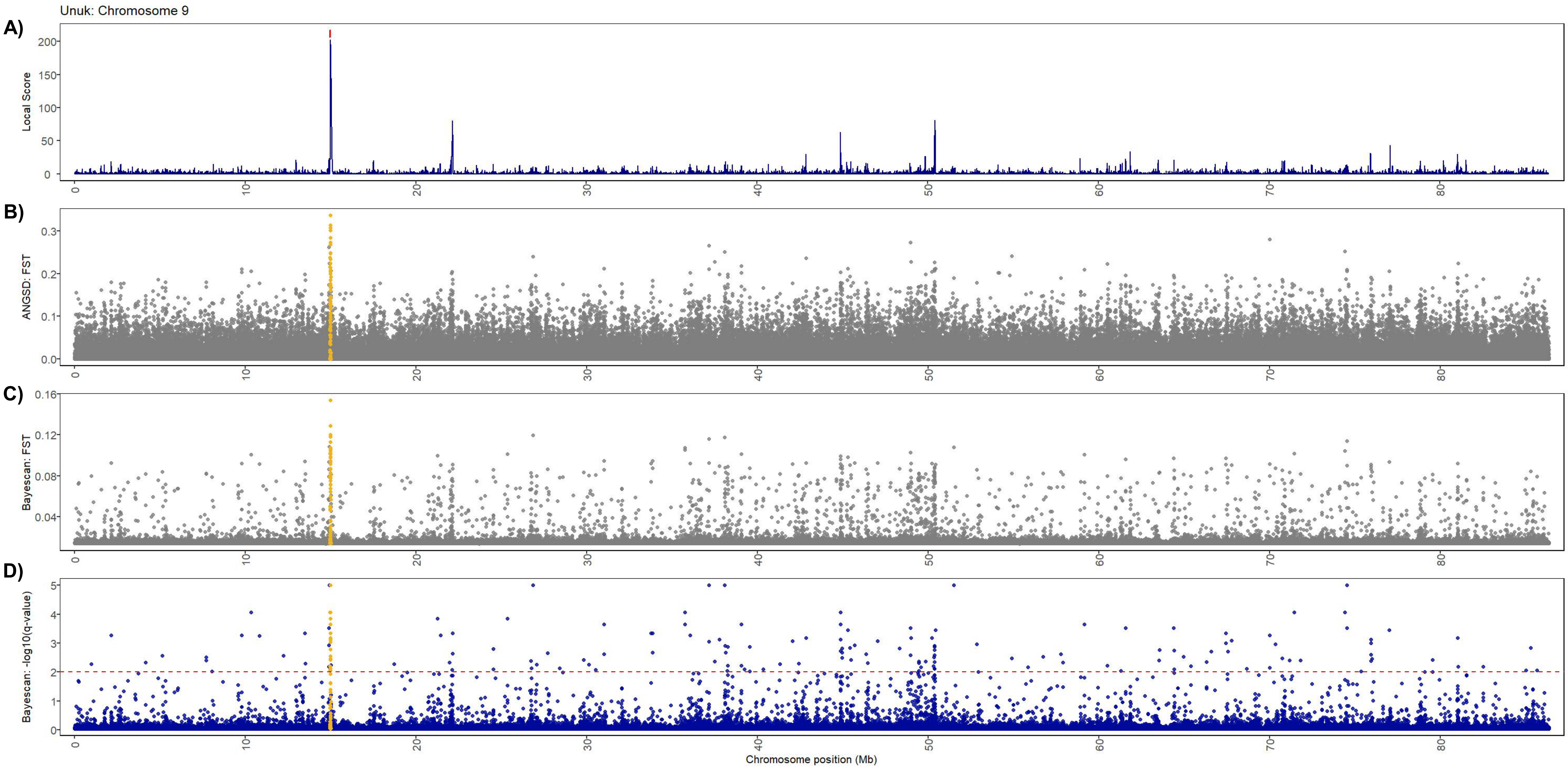

Manhattan plots for Unuk H-W comparison on chromosome 9. A) Local score plot with the red rectangle signifying an outlier peak identified through local score; B) FST calculated in ANGSD; C) FST calculated in Bayescan; D) –log(q-value) calculated in Bayescan with the red dashed line signifying the cutoff for outlier loci expected to be under selection. The yellow points in panels B – D are loci within the local score outlier peak boundaries.

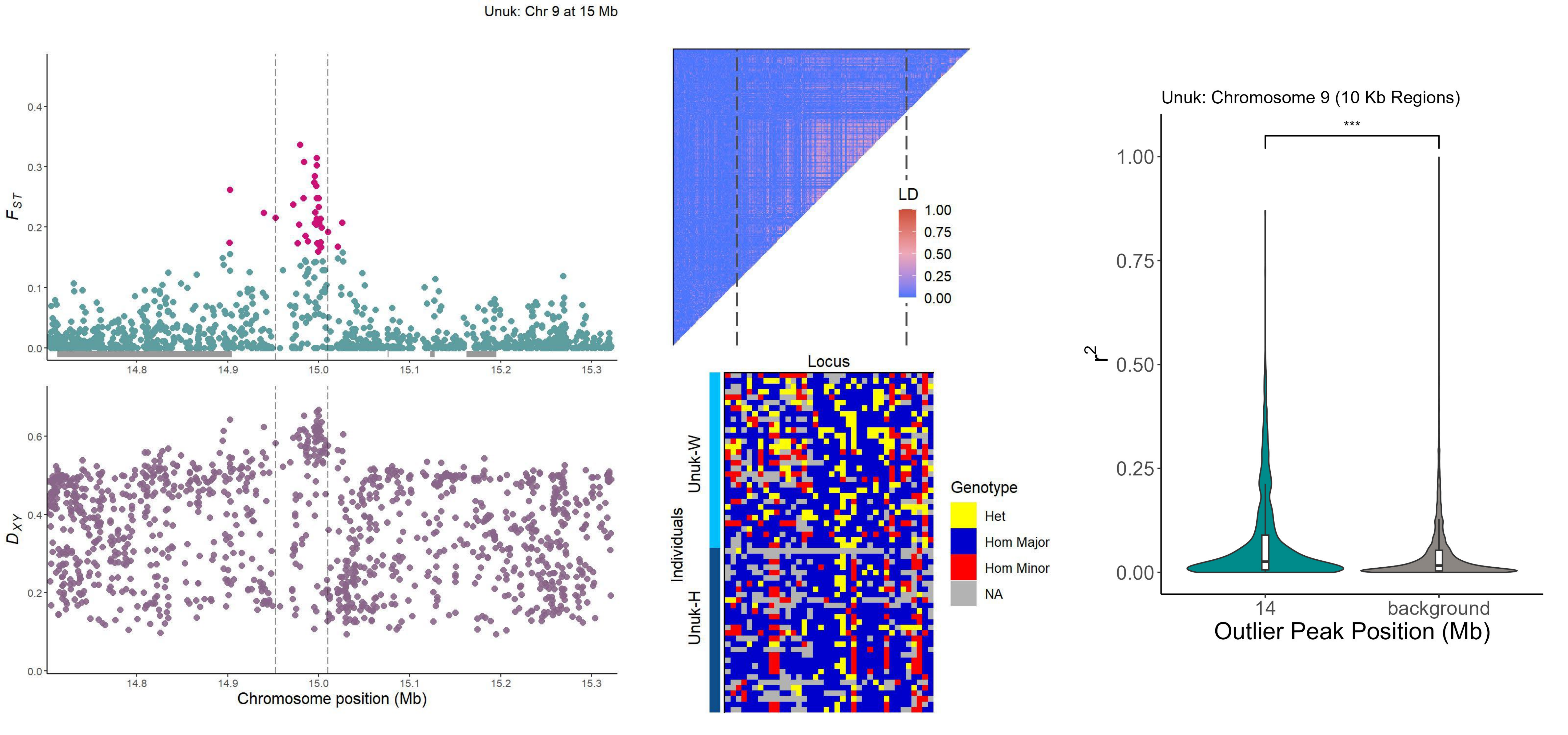

Unuk: Chr 9 at 15 Mb
F_{ST}
0.0
0.1
0.2
0.3
0.4
14.8
14.9
15.0
15.1
15.2
15.3
D_{XY}
0.0
0.2
0.4
0.6
Chromosome position (Mb)
LD
1.00
0.75
0.50
0.25
0.00
Locus
Individuals
Unuk-W
Unuk-H
Genotype
Het
Hom Major
Hom Minor
NA
Unuk: Chromosome 9 (10 Kb Regions)
***
r^2
1.00
0.75
0.50
0.25
0.00
14
background
Outlier Peak Position (Mb)
